# A systematic evaluation of single-cell RNA-sequencing imputation methods

**DOI:** 10.1186/s13059-020-02132-x

**Published:** 2020-08-27

**Authors:** Wenpin Hou, Zhicheng Ji, Hongkai Ji, Stephanie C. Hicks

**Affiliations:** Department of Biostatistics, Johns Hopkins Bloomberg School of Public Health, 615 North Wolfe Street, Baltimore, 21205 MD USA

**Keywords:** Gene expression, Single-cell RNA-sequencing, Imputation, Benchmark

## Abstract

**Background:**

The rapid development of single-cell RNA-sequencing (scRNA-seq) technologies has led to the emergence of many methods for removing systematic technical noises, including imputation methods, which aim to address the increased sparsity observed in single-cell data. Although many imputation methods have been developed, there is no consensus on how methods compare to each other.

**Results:**

Here, we perform a systematic evaluation of 18 scRNA-seq imputation methods to assess their accuracy and usability. We benchmark these methods in terms of the similarity between imputed cell profiles and bulk samples and whether these methods recover relevant biological signals or introduce spurious noise in downstream differential expression, unsupervised clustering, and pseudotemporal trajectory analyses, as well as their computational run time, memory usage, and scalability. Methods are evaluated using data from both cell lines and tissues and from both plate- and droplet-based single-cell platforms.

**Conclusions:**

We found that the majority of scRNA-seq imputation methods outperformed no imputation in recovering gene expression observed in bulk RNA-seq. However, the majority of the methods did not improve performance in downstream analyses compared to no imputation, in particular for clustering and trajectory analysis, and thus should be used with caution. In addition, we found substantial variability in the performance of the methods within each evaluation aspect. Overall, MAGIC, kNN-smoothing, and SAVER were found to outperform the other methods most consistently.

## Background

Recent advances in high-throughput technologies have been developed to measure gene expression in individual cells [1–5]. In contrast to bulk RNA-sequencing (RNA-seq), a distinctive feature of data measured using single-cell RNA-sequencing (scRNA-seq) is the increased sparsity, or fraction of observed “zeros,” where a zero refers to no unique molecular identifiers (UMIs) or reads mapping to a given gene in a cell [6–9]. These observed zeros can be due to biological (relevant or nuisance) fluctuations in the measured trait or technical limitations related to challenges in quantifying small numbers of molecules. Examples of the latter include mRNA degradation during cell lysis or variation by chance of sampling lowly expressed transcripts [10]. The word *dropout* [6–8] has been previously used to describe both biological and technical observed zeros, but the problem with using this catch-all term is it does not distinguish between the types of sparsity [10].

To address the increased sparsity observed in scRNA-seq data, recent work has led to the development of “imputation” methods, in a similar spirit to imputing genotype data for genotypes that are missing or not observed. However, one major difference is that in scRNA-seq standard transcriptome reference maps such as the Human Cell Atlas [11] or the Tabula Muris Consortium [12] are not yet widely available for all species, tissue types, genders, and so on. Therefore, the majority of imputation methods developed to date do not rely on an external reference map.

These imputation methods can be categorized into three broad approaches [10]. The first group are imputation methods that directly model the sparsity using probabilistic models. These methods may or may not distinguish between biological and technical zeros, but if they do, they typically impute gene expression values for only the latter. A second approach adjusts (usually) all values (zero and non-zero) by smoothing or diffusing the gene expression values in cells with a similar expression profiles identified, for example, using neighbors in graph. The third approach first identifies a latent space representation of the cells, either through low-rank matrix-based methods (capturing linear relationships) or deep-learning methods (capturing non-linear relationships), and then reconstructs the observed expression matrix from the low-rank or estimated latent spaces, which will no longer be sparse. For the deep-learning approaches, such as variational autoencoders, both the estimated latent spaces and the “imputed” data (i.e., reconstructed expression matrix) can be used for downstream analyses, but otherwise only the imputed data is typically provided for downstream analyses.

**Fig. 1:**
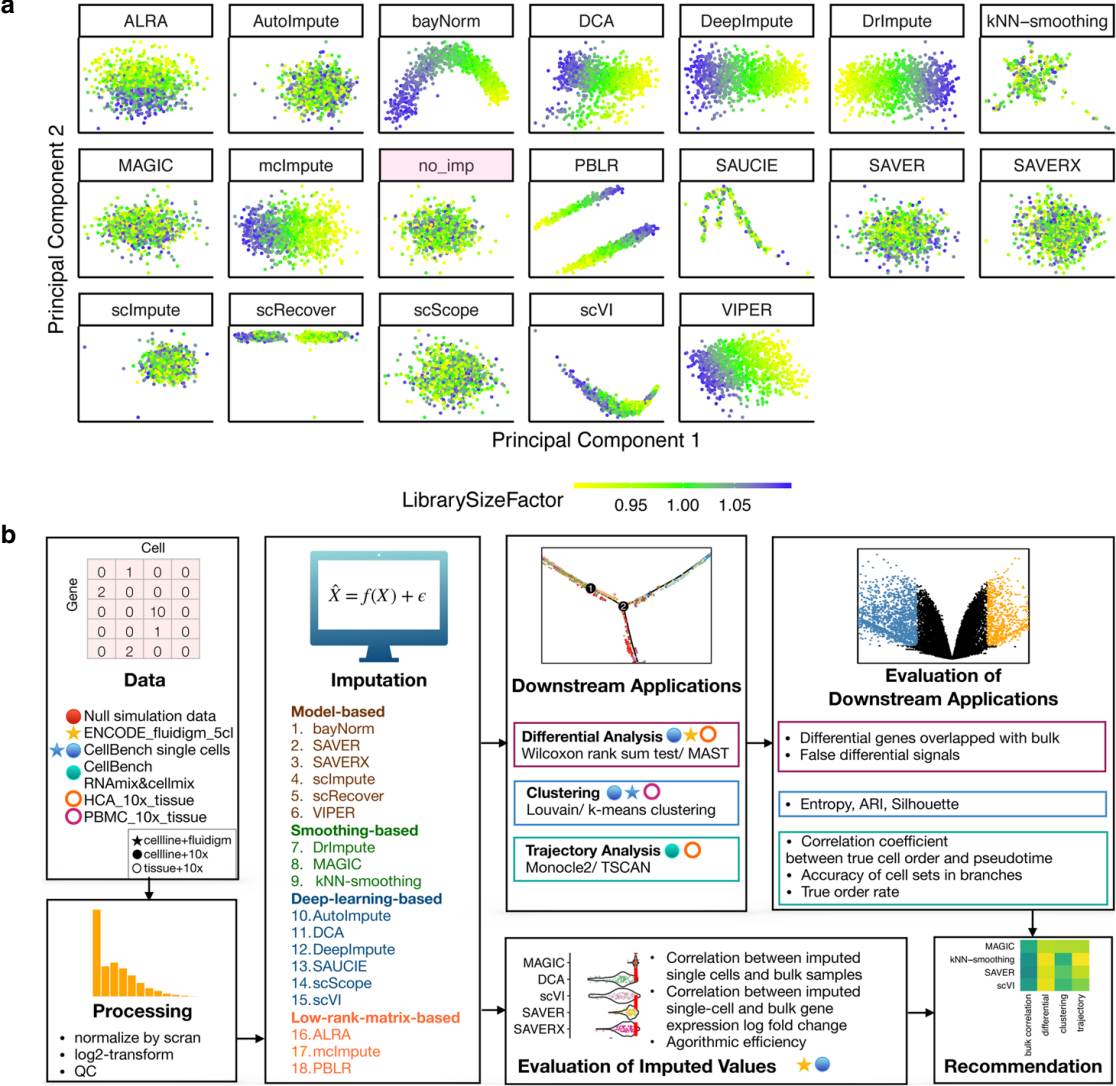
Motivation and overview of benchmark evaluation of scRNA-seq imputation methods. **a** Dimension reduction results after applying principal components analysis (PCA) from either no imputation method (*no_imp* highlighted in red) or the 18 imputation methods using the *null simulations* data where no structural pattern is expected. The color represents the simulated library size (defined as the total sum of counts across all relevant features) for each cell. **b** An overview of the benchmark comparison evaluating 18 scRNA-seq imputation methods

Due to these recent and concurrent development, evaluations and comparisons between scRNA-seq imputation methods have been limited or restricted to a subset of imputation methods and downstream applications [13–16]. Furthermore, imputation methods can require varying types of raw or processed data as input, may rely on different methodological assumptions, and may be appropriate for only certain scRNA-seq experimental protocols, such as UMI-based [1–3] or full-length [4, 17] transcript methods. Given these differences, the performance of these methods has been shown to vary in the evaluations to-date. For example, one study found imputation methods can introduce false signals when identifying differentially expressed genes with model-based methods outperforming smoothing-based methods, in particular for genes with a small effect size (log2 fold change) [14]. Another study found imputation methods can introduce spurious correlations between imputed expression and total UMI counts [15]. Alternatively, others have shown spurious structural patterns in low dimensional representations of imputed data [14, 18], which we also find in data where we expect no structural patterns in the data, but patterns associated with library size emerge in the imputed data (Fig. 1a, Additional file 1: Figure S1). In contrast, others have found a subset of imputation methods to be helpful to estimate library size factors for normalization of sparse scRNA-seq data [16]. Therefore, the answer to the question of which methods *can*, let alone *should*, be used for a particular analysis is often unclear.

To address this gap, we performed a systematic benchmark comparison and evaluation of 18 state-of-the-art scRNA-seq imputation methods (Fig. 1b). Specifically, we evaluated (1) model-based imputation methods (bayNorm [19], SAVER [20], SAVER-X [21], scImpute [22], scRecover [23], VIPER [24]), (2) smooth-based imputation methods (DrImpute [25], MAGIC [26], kNN-smoothing [27]), and (3) data reconstruction methods either using deep-learning methods (AutoImpute [28], DCA [29], DeepImpute [30], SAUCIE [31], scScope [32], scVI [33]) or low-rank matrix-based methods (ALRA [34], mcImpute [35], PBLR [36]). Compared to existing benchmark evaluations of imputation methods, our benchmark is more comprehensive in terms of (i) the number of imputation methods considered (18 imputation methods compared to 3–8 in previous benchmarks [13, 14, 18]), (ii) the types of tissues considered (both cell lines and tissues), (iii) the number of downstream analyses considered (four applications compared to just one, for example, differential expression [14]), and (iv) different types of experimental protocols considered (both droplet- and plate-based protocols as opposed to just plate-based [13]), all of which is described in Additional file 2: Table S1. Throughout, we use teletype (or monospace) font when referring to specific software packages and *italicized* font when referring to datasets. While many of the imputation methods used raw scRNA-seq UMI or read counts as input, a subset of these methods required normalized (or log-transformed normalized) counts. In the latter case, we used the scRNA-seq pooling normalization method [37] implemented in the scran [38] R/Bioconductor [39, 40] package, which has been previously shown to outperform other scRNA-seq normalization methods in full-length and UMI-based methods [16, 18]. We also included a baseline comparison of “no imputation,” which is the raw scRNA-seq counts that have been adjusted for only library size with scran [37] normalization. While there are more methods available that could be used for imputation, we only included methods in our benchmark that (i) were originally designed (or specified by the authors) to be used as an imputation method, (ii) included software for users to download and run locally, and (iii) did not need external pieces of information (e.g., a network or an external reference map) and used scRNA-seq counts (raw or normalized) as input.

In this paper, we first evaluate the performance of the imputation method themselves on their ability to recover true expression values by comparing the similarity between imputed cell profiles and bulk samples in a homogeneous population of cells. Then, we investigate the performance of the imputation methods in downstream analyses including differential expression analysis, unsupervised clustering, and trajectory analysis. In addition to simulated data, we used two types of real single-cell data: cell lines and tissues measured across experimental platforms, including full-length and UMI methods using plate-based and droplet-based protocols. We summarize our results and provide a key set of recommendations for users and investigators to navigate the current space of scRNA-seq imputation methods.

## Results

### Similarity between bulk RNA-seq and imputed scRNA-seq data

**Fig. 2:**
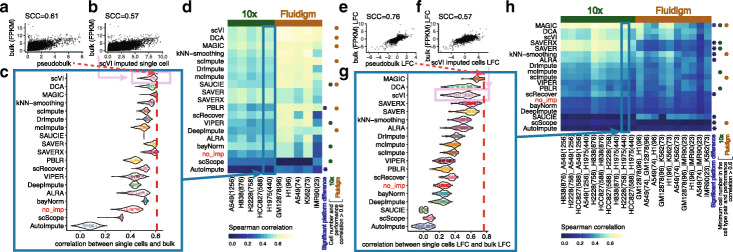
Similarity between bulk and imputed single-cell expression data in cell lines. **a** For the H1975 cell line, a scatter plot of the scran normalized [37] log2-transformed scRNA-seq cell profiles (*N* = 440) averaged across all cells (“pseudobulk”) with that in a bulk RNA-seq profile with the Spearman’s correlation coefficient (SCC). **b** For each cell, we also calculated the SCC between an imputed cell’s profile (e.g., using scVI) and the bulk RNA-seq profile. **c** Distribution of correlations between bulk profiles and single-cell profiles (imputed or not imputed—i.e., *no_imp*) across all cells in the H1975 cell line dataset. The red dotted line represents the estimated SCC ($\hat {\rho }$ = 0.61) shown in **a**. Here, we use the pseudobulk as a reference for an upper bound in performance. While the correlation between bulk and pseudobulk is higher than between bulk and an imputed cell’s profile, the imputed profiles are still useful because pseudobulk ignores cell variability. The methods are ordered in the same order as **d** for comparison. **d** A heatmap of the median correlation for each imputation method and each dataset across two experimental platforms (five datasets from the 10x Genomics platform and five datasets from the Fluidigm platform with the number of cells in each dataset in parentheses). The rows are sorted by first averaging the median correlations across datasets within each platform and then averaging across platforms. The asterisks are used to denote the methods with significant platform difference, defined as the Benjamini-Hochberg adjusted *p* values (i.e., FDR) < 0.05 from two-sample *t*-tests that evaluate whether the SCCs have equal mean between the two (10x and Fluidigm) platforms, and the relative performance change $|\overline {SCC}_{10x}-\overline {SCC}_{fluidigm}| / max(\overline {SCC}_{10x},\overline {SCC}_{fluidigm})$ is greater than 25%. Filled circles (brown: Fluidigm, green: 10x) indicate methods for which the imputation performance (values in a row) and the number of cells in a dataset show high correlation (Spearman correlation ≥ 0.6). **e**–**h** Similar to **a**–**d** except, for any two cell types, the SCC is calculated comparing the difference (log fold change or LFC) in two bulk cell type profiles compared to two scRNA-seq cell type profiles. The average number of cells across two cell lines is shown in parentheses. The minimum cell number in the cell type pair is used for computing the cell number -performance correlation

To evaluate an imputation method’s ability to recover biological expression observed in bulk RNA-seq data, we assessed the similarity between bulk and imputed scRNA-seq data using cell lines (Fig. 2). Here, we focused on cell lines since they are less heterogeneous than tissues and have well-defined bulk expression profiles. The imputed values from the scRNA-seq data were evaluated in two settings.

In the first evaluation, we directly compared imputed scRNA-seq profiles from cell lines to a bulk RNA-seq profile from the same cell lines (Fig. 2a–d). The test data include 10x Genomics UMI-based scRNA-seq data for five CellBench [18] cell lines (*sc_10x_5cl*) and Fluidigm C1 plate-based scRNA-seq read count data for five ENCODE cell lines (*ENCODE_fluidigm_5cl*). Using the rank-based Spearman correlation coefficient (SCC) [41] between the imputed scRNA-seq profile and bulk profile, the majority of imputation methods (16 out of 18) improved the correlation compared to not imputing. Methods such as SAVER and SAVER-X (without pre-training) performed well with 10x Genomics UMI count data (ranked 2 and 3 out of 18), but their performance gain was not as pronounced with read count data from the plate-based Fluidigm platform (ranked 12 and 13 out of 18) (Fig. 2d). We note that this difference in performance is expected as SAVER and SAVER-X have assumed a negative binomial distribution for UMI data [20, 21]. UMI count data have been shown to follow a negative binomial distribution as opposed to read count data which have been shown to follow a zero-inflated negative binomial distribution [42–45]. Within each platform, we found that the methods scVI, DCA, and MAGIC performed better compared to the other methods, and this was consistent in both the Fluidigm and 10x platforms (Fig. 2d). When comparing across platforms, PBLR and AutoImpute showed significant cross-platform performance differences (two-sample *t*-tests at a false discovery rate (FDR) < 0.05 and relative performance change $ |\overline {SCC}_{10x}-\overline {SCC}_{fluidigm}| / max(\overline {SCC}_{10x},\overline {SCC}_{fluidigm}) > 25\%$). For both methods, the correlation between the imputed single-cell profile and bulk profile was higher in the Fluidigm platform than in the 10x platform (Fig. 2d).

In the second evaluation, we assessed an imputation method’s ability to preserve the difference on the log scale between two cell type profiles (i.e., two cell lines) by comparing the difference in two single-cell cell type profiles to the difference in two bulk cell type profiles (Fig. 2e–h). Compared to Fig. 2a–d, the majority, though a smaller set, of imputation methods (13 out of 18) preserved the cell type difference better than no imputation. The imputation methods MAGIC, DCA, and scVI resulted in the highest correlation using both UMI and non-UMI plate-based protocols (Fig. 2h). Similar to Fig. 2d, SAVER and SAVER-X (without pretraining) resulted in the much higher correlation using UMI count data than using non-UMI plate-based data. When comparing across platforms, 9 out of 18 imputation methods showed significant cross-platform performance differences (two-sample *t*-tests FDR < 0.05, relative performance change > 25*%*) (Fig. 2h). Among these methods, AutoImpute and SAUCIE showed higher correlation between the imputed expression difference and the bulk expression difference in the Fluidigm platform, while the other methods (MAGIC, SAVERX, SAVER, kNN-smoothing, ALRA, scRecover, scScope) showed higher correlation in the 10x platform.

Finally, the performance of some imputation methods were found to be affected by the number of cells in the dataset. For data generated using the same platform, we observed that a smaller cell number is associated with a smaller imputed value-bulk correlation for these methods (Fig. 2d, h: green and brown dots on the right of the heatmaps; and Additional file 3: Table S2). For example, for Fluidigm datasets in Fig. 2d, IMR90 with 23 cells showed smaller correlation compared to the other plate-based datasets with more cells. For methods such as scVI, DCA, MAGIC, scImpute, SAUCIE, PBLR, and DeepImpute, the Spearman correlation between the cell number and performance (imputation-bulk SCC) across the Fluidigm datasets was higher than 0.6 (Fig. 2d).

### Impact of scRNA-seq imputation on identifying differentially expressed genes

Next, we evaluated the impact of imputation on the downstream analysis of identifying differentially expressed genes (DEGs). We intentionally designed our evaluation to primarily rely on empirical analyses of real data in order to preserve gene-gene correlations. In these empirical analyses, the ground truth was not completely known. Thus, our evaluation could not explicitly calculate sensitivity and specificity as previous studies [14, 15]. However, we preferred empirical evaluation over simulation. This is because some imputation methods model the expression levels for one gene based on the expression levels of other genes, such as with SAVER and SAVER-X, and simulation or spike-in studies in which ground truth is known but the gene-gene correlation is disrupted would unfairly disfavor such methods. Also, modeling the complex gene-gene correlation in real data via simulation and spike-in studies is difficult. We considered two methods for differential expression (DE) analysis (namely MAST [46] and Wilcoxon rank-sum test [47] abbreviated as Wilcoxon), because we wanted to assess whether the performance difference among the imputation methods in this section was consistent regardless of the choice of DE method or whether it was due to the choice of the DE method. While MAST uses a parametric model developed specifically for single-cell data, the Wilcoxon test is a non-parametric test commonly used for both bulk and single-cell data.

**Fig. 3:**
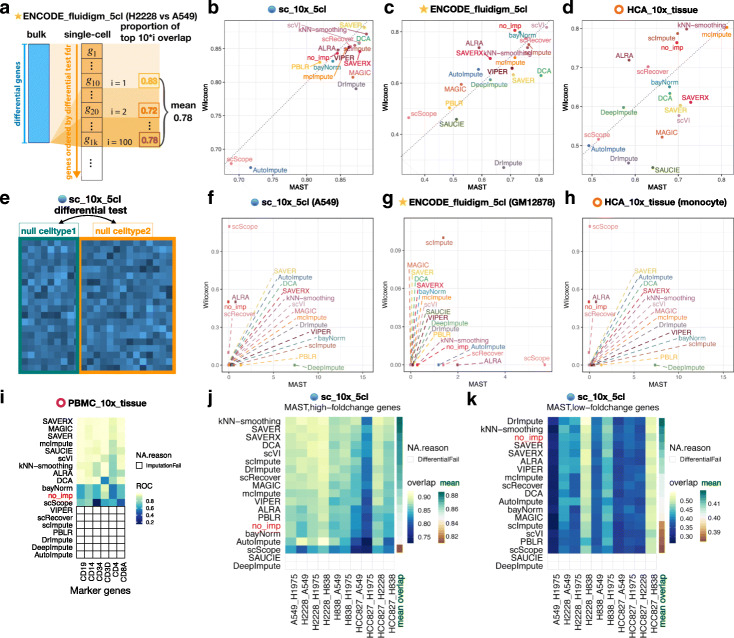
Impact of imputation methods on differential expression analysis. For each imputation method, we performed three gene-level analyses. **a** Schematic view of evaluating differentially expressed genes (DEGs) using the overlap between bulk RNA-seq and scRNA-seq. **b**–**d** Proportion of overlap between bulk and single-cell DEGs identified using either MAST (*x*-axis) or Wilcoxon rank-sum test (*y*-axis). Note that *“cl”* in the names of datasets means “cell line.” **e** Schematic view of a null DE analysis. **f**–**h** Number of false positive DEGs averaging across all settings identified by MAST (*x*-axis) or Wilcoxon rank-sum test (*y*-axis) in null differential analyses. **i** Heatmap of area under a receiver operating characteristic (ROC) curve values when using the expression level of a marker gene (e.g., CD19) to predict a cell type (e.g., B cell or not) using UMI-based sorted PBMC cell types. For some imputation methods, no imputed values were returned. They are denoted as “ImputationFail”. **j**, **k** Using a UMI-based scRNA-seq dataset from cell lines (*sc_10x_5cl*), a heatmap showing the percentage of the overlap between bulk and single-cell DEGs identified using MAST stratified by genes with high (top 10%) or low (bottom 10%) log-fold changes. The color bar on the last column shows the mean overlap across all comparison for each method. If MAST failed to identify DEGs from the imputed profiles of any method in any dataset, we denoted it as “DifferentialFail.” Please refer to Additional file 1: Figure S5 for the Wilcoxon results

In the first analysis, we performed a DE enrichment analysis (Fig. 3a). We treated genes identified as differentially expressed in bulk RNA-seq data as a “gold standard” similar to previous studies [48]. Then, we calculated the overlap of DEGs between the bulk data and DEGs identified from scRNA-seq data between the same two cell types using MAST and Wilcoxon (Fig. 3b–d, Additional file 1: Figure S2a-g). Note that the analyses presented in Fig. 3b–d are different from those presented in Fig. 2e–h. Figure 2e–h only consider the log-fold change (LFC) between two cell types. In contrast, in Fig. 3b–d we also estimate the uncertainty of LFC (due to cell-to-cell variability) and take it into account in order to rank genes, similar to what one would do in hypothesis testing.

Using UMI-based 10x Genomics scRNA-seq data from five cell lines (*sc_10x_5cl*), we found that 12 and 10 out of the 18 methods outperformed no imputation using MAST and Wilcoxon, respectively (Additional file 1: Figure S2b, c). Among them, kNN-smoothing, SAVER, and SAVER-X (without pretraining) had the highest overlap of single-cell DEGs with bulk DEGs using MAST, but SAVER, kNN-smoothing, and scVI performed the best when using Wilcoxon (Additional file 1: Figure S2b, c). While MAGIC performed well when calculating log-fold changes (Fig. 2h), we found that MAGIC’s performance dropped when taking into account the estimates of uncertainty (Additional file 1: Figure S2B,C). Further investigation shows that the estimated gene-specific standard deviations from the MAGIC imputed values were much smaller compared to the estimated standard deviations from other imputation methods (Additional file 1: Figure S3). In turn, the estimated standard errors for the log-fold changes using MAGIC were small, leading to a wide test-statistic distribution and a *p* value distribution skewed toward small *p* values (Additional file 1: Figure S4). This suggests that MAGIC could have inaccurately estimated cell variability, which reduced its gene ranking performance.

Using the plate-based read count data from five ENCODE cell lines (*ENCODE_fluidigm_5cl*), scVI performed best using either MAST or Wilcoxon (Additional file 1: Figure S2d-e). However, only 7 and 1 out of the 18 methods increased the overlap of DEGs compared to no imputation when using MAST and Wilcoxon tests, respectively.

We also compared methods using the UMI-based scRNA-seq bone marrow tissue data (*HCA_10x_tissue*) in the 10x Genomics platform. Because cells in this dataset were unsorted, we first computationally labeled cell types using bulk RNA-seq data (described in “Methods” section). Here, we found that mcImpute and kNN-smoothing were in the top three either using MAST or Wilcoxon (Additional file 1: Figure S2f-g). Similar to using the *ENCODE_fluidigm_5cl* dataset, we found a small number of methods (6 and 3 out of 18 methods using MAST and Wilcoxon, respectively) that had a higher overlap between single-cell and bulk DEGs compared to no imputation.

To summarize this DE enrichment analysis across the three datasets (*sc_10x_5cl*, *ENCODE_fluidigm_5cl*, *HCA_10x_tissue*) and two DE methods (MAST and Wilcoxon), broadly we found kNN-smoothing (a smoothing-based method) and scVI (a deep-learning method) had the highest overlap with bulk DEGs (Fig. 3b–d), and they both most consistently outperformed no imputation (in 5 out of 6 test settings in Additional file 1: Figure S2b-g). The model-based methods SAVER and SAVER-X were among the top performers with UMI-based datasets, and they outperformed no imputation in 3 out of the 4 UMI-based test settings (Additional file 1: Figure S2b, c, f, g).

Next, we performed a null DE analysis using cells from a homogeneous population where we expect no DEGs after correction for multiple testing. We selected three cell types, namely the A549 cell line from the *sc_10x_5cl* dataset, GM12878 from *ENCODE_fluidigm_5cl*, and the monocytes from *HCA_10x_tissue*. For each cell type, we randomly split cells into two groups (Fig. 3e) with varying sizes ranging from *N* = 10 to 500 cells per group, imputed the expression values and identified DEGs (Fig. 3f–h, Additional file 1: Figure S2h-n). Broadly, we found the majority of methods perform well in this analysis (specifically most methods are close to the (0,0) coordinate in Fig. 3f–h), but for the methods that identified false positive DEGs, we found no consistency in which types of imputation methods identified false positive DEGs as there were examples in all types of imputation methods (model-based, smoothing-based, and data reconstruction based) that reported false positive DEGs (Additional file 1: Figure S2i-n). We also observed that imbalanced group sizes of cells when identifying DEGs resulted in more imputation methods reporting false positives using both MAST and Wilcoxon (Additional file 1: Figure S2i-j).

To complement the enrichment and null DE analysis, we also asked if the imputed expression of known cell-type-specific marker genes can correctly predict cell type. Using a UMI-based and FACS sorted peripheral blood mononuclear cell (PBMC) dataset [3] (*PBMC_10x_tissue*) and known PBMC marker genes highly expressed in purified PBMC cell types (CD19 for B cells; CD14 for monocytes; CD34 for CD34 + cells; CD3D for CD4 T helper cells, cytotoxic T cells, memory T cells, naive cytotoxic T cells, naive T cells, regulatory T cells; CD8A for cytotoxic T cells and naive cytotoxic T cells), we evaluated the performance of predicting a cell type (e.g., B cell) based on the expression of a marker gene (e.g., CD19) (Fig. 3i). We estimated the area under the ROC (AUROC) curve where the expression of the marker gene (e.g., CD19 expression) is the predictor and the true cell type (e.g., B cell or not) is the label (for more details see “Methods” section). Among the methods that returned imputed values, we found that 10 out of 11 imputation methods (that returned imputation values) produced higher AUROC compared to no imputation (Fig. 3i). Specifically, SAVER-X, MAGIC, and SAVER were the top three methods with this UMI-based scRNA-seq data.

We further evaluated the impact of the magnitude of DE (i.e., effect size) on an imputation method, which was previously shown to be important for the performance of imputation methods in the context of identifying DEG [14]. Using the *sc_10x_5cl* dataset with five cell lines, we compared the cell lines pairwise and stratified genes into high and low LFC using the bulk RNA-seq data, where high (or low) LFC genes were defined as the top 10% (or bottom 10%) genes based on absolute values of LFC. Interestingly, when the magnitude of DE was large, the overlap between the bulk and single-cell DEGs for most imputation methods (13 out of 16 methods that returned imputed values) was higher compared to no imputation (Fig. 3j). However, when the magnitude of DE was small, only 2 out of the 16 methods increased the overlap between the bulk and single-cell DEGs compared to no imputation, suggesting that most imputation methods may have smoothed away small differential signals (Fig. 3k). In Fig. 3k, the DEG overlap was highly variable across datasets, with some datasets showing larger overlap compared to other datasets regardless of imputation methods. This is because different datasets have different signal abundance levels, and methods tend to show a larger imputation-bulk overlap in datasets with more bulk DE genes compared to datasets with fewer bulk DE genes (Additional file 1: Figure S5a). In real applications, the signal abundance is often determined by the underlying biology rather than determined by users. Thus, the primary goal of this analysis is to compare different imputation methods on the same dataset rather than studying dataset variability. To more clearly show the method difference conditional on the same dataset, we further ranked all methods within each dataset and compared the ranks. The conclusion remained similar: most imputation methods improved analyses for DE genes with large LFC over no imputation but did not improve for genes with small LFC (Additional file 1: Figure S5b, c). The above analyses were performed with MAST, but we found consistent results using the Wilcoxon test (Additional file 1: Figure S5d, e).

### Impact of scRNA-seq imputation on unsupervised clustering

Unsupervised clustering is another common downstream analysis with scRNA-seq data to empirically define groups of cells with similar expression profiles [40]. Here, we assessed the impact of the 18 imputation methods on unsupervised clustering, specifically using *k*-means [49] and Louvain clustering [50]. Similar to our motivation for using two DE methods (MAST and Wilcoxon), we considered two unsupervised clustering methods in this section in order to assess whether the performance difference among the imputation methods was due to the choice of the clustering method or if the relative performance of imputation methods was consistent regardless of the choice of clustering method. However, instead of only considering the imputed gene expression profiles from the 18 imputation methods, we also considered the three latent spaces from scVI, scScope, and SAUCIE (for a total of 21 “methods”) for unsupervised clustering and inferring pseudotemporal trajectories. Clustering was performed both on the top principal components of the imputed data from the 18 methods and on the three latent spaces directly provided by scVI, scScope, and SAUCIE. We used four metrics to assess the clustering performance: the median Silhouette index, adjusted Rand index (ARI) [51], entropy of cluster accuracy (*H*_*acc*_), and entropy of cluster purity (*H*_*pur*_). The last three were also used by and described in Tian et al. (2019) [18]. The Silhouette index measures consistency within clusters (or how similar an observation is to its own cluster compared to other clusters). The last three metrics assess the similarity of predicted cluster labels to a known ground truth, and they have been shown to have good correlation with each other [18]. The primary difference between the last two is that *H*_*acc*_ measures the diversity (or accuracy) of the true group label within each cluster assigned by the clustering method, while *H*_*pur*_ measures the diversity (or purity) of the predicted cluster labels within each of the true groups. We scaled ARI to range between 0 and 1, and Silhouette index ranges between − 1 and 1. In both cases, a higher score represents a better performance. *H*_*acc*_ and *H*_*pur*_ range between 0 and a number larger than one with a lower score representing a better performance.

**Fig. 4:**
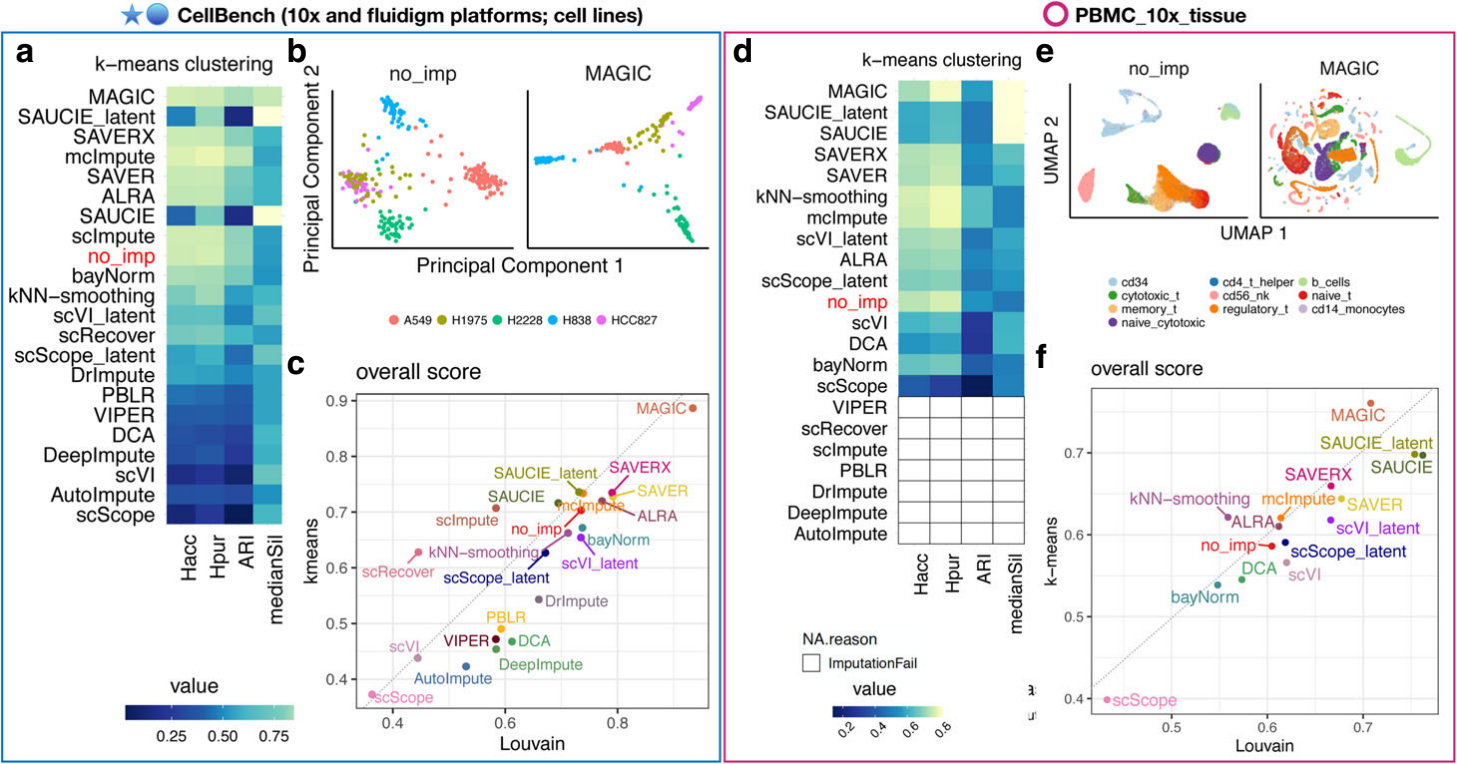
Impact of imputation methods on unsupervised clustering analysis. **a** Heatmap of four performance metrics—entropy of cluster accuracy (*H*_*acc*_), entropy of cluster purity (*H*_*pur*_), adjusted Rand index (ARI), and median Silhouette index—averaged across seven datasets from CellBench [18]. Each metric shows the average performance across 7 datasets in CellBench. To compare imputation methods across metrics, the metrics were re-scaled to between 0 and 1 and the order of *H*_*acc*_ and *H*_*pur*_ were flipped to where a higher standardized score translates to better performance. Imputation methods (rows) are ranked by the average performance between the mean of the first three metrics (*H*_*acc*_,*H*_*pur*_ and ARI) and the fourth metric (*medianSil*). **b** Dimension reduction results after applying PCA to the *sc_celseq2_5cl_p1* data with no imputation (left) and with imputation using MAGIC (right). The colors are the true group labels. **c** Overall score (or average of the four performance metrics) for Louvain clustering (*x*-axis) and *k*-means clustering (*y*-axis). **d**–**f** Same as **a**–**c** except using the scRNA-seq dataset of ten sorted peripheral blood mononuclear cell (PBMC) cell types from 10x Genomics [3] (*PBMC_10x_tissue* dataset). White areas with black outline in **d** indicate that the imputation methods did not return output after 72 h. Also, **e** uses UMAP components [52] instead of principal components. Please refer to Additional file 1: Figures S6, S7, S9 for Louvain clustering results and metrics in each dataset and Additional file 1: Figure S8 for UMAPs of other methods

We applied each imputation method to seven datasets from CellBench [18] (Additional file 4: Table S3, the “Methods” section), a data compendium consisting of both UMI-based 10x Genomics, Drop-seq, and plate-based scRNA-seq data for benchmarking analysis methods, and then applied *k*-means clustering (Fig. 4a–c, Additional file 1: Figure S6). A similar evaluation using the CellBench data was performed in Tian et al. [18] who evaluated three scRNA-seq imputation methods (kNN-smoothing [27], DrImpute [25], and SAVER [20]) with five unsupervised clustering methods. Here, we expanded their analysis to include 18 scRNA-seq imputation methods and 3 latent space outputs from scVI, scScope, and SAUCIE—for a total of 21 “methods.” Our primary goal here is to evaluate the imputation methods rather than clustering methods.

Broadly, using *k*-means clustering 8 out of 21 methods (MAGIC, SAUCIE_latent, SAVER-X, mcImpute, SAVER, etc.) improved clustering results compared to no imputation in these data (Fig. 4a). Overall, using the latent spaces for scVI, scScope and SAUCIE was better than using their imputed expression values (e.g., observations sampled from the posterior distribution using scVI). We illustrate how individual cells cluster along the first two principal components using an example dataset from CellBench (*sc_celseq2_5cl_p1*) with no imputation and with imputation using MAGIC (Fig. 4b). Using Louvain clustering yielded similar results (Fig. 4c, Additional file 1: Figure S7) although the top methods were slightly different (MAGIC, SAVER, SAVER-X, ALRA, mcImpute, and bayNorm).

We further compared imputation methods using the scRNA-seq dataset of ten sorted PBMC cell types from 10x Genomics [3] (*PBMC_10x_tissue*) using both *k*-means clustering and Louvain clustering. For the imputation methods that successfully returned imputed values, 10 methods (MAGIC, SAUCIE_latent, SAUCIE, SAVER-X, SAVER, etc.) outperformed no imputation (Fig. 4d). In Fig. 4e, we show how the sorted PBMC cells cluster along the UMAP components [52] with no imputation and with imputation using MAGIC (other methods see Additional file 1: Figure S8). Again, the results between *k*-means and Louvain clustering were similar (Fig. 4f, Additional file 1: Figure S9) though the top methods when using Louvain clustering were slightly different (SAUCIE, SAUCIE_latent, MAGIC, SAVER, SAVER-X). We provide UMAP plots of the cells colored by true cell types and the clustering results from both *k*-means and Louvain clustering in Additional file 1: Figure S8.

### Impact of scRNA-seq imputation on inferring pseudotemporal trajectories

We also evaluated the impact of imputation methods on inferring cells’ pseudotemporal trajectories. In contrast to clustering analysis which used data with distinct cell types to evaluate clustering, here we used datasets in which cells had a continuum of transcriptomic profiles (e.g., cell differentiation) to assess if imputation methods could recover continuous biological processes. Analogous to the section above, we applied methods to infer trajectories on the top principal components. We also inferred trajectories using the latent spaces directly provided by scVI, scScope, and SAUCIE, similar to clustering analysis.

First, we applied imputation methods to six RNA mixture and cell mixture datasets from CellBench [18] followed by using two different trajectory analysis methods, Monocle 2 [53] and TSCAN [54]. In these data, the true trajectories of cells were known. They were used to evaluate the impact of imputation methods on the ability to infer trajectories. We considered two trajectory inference methods in order to assess whether the performance difference among the imputation methods was due to the choice of the trajectory inference method or if the relative performance of imputation methods was consistent regardless of the choice of trajectory inference method. A similar evaluation using CellBench data was performed by Tian et al. [18] who evaluated three scRNA-seq imputation methods (kNN-smoothing [27], DrImpute [25], and SAVER [20]) with five trajectory inference methods. Here, we expanded their analysis to include not only the imputed gene expression profiles from the 18 imputation methods, but also the three latent spaces from scVI, scScope, and SAUCIE (for a total of 21 “methods”). While we include two trajectory inference methods, we note our primary focus is to evaluate the imputation methods. The performance metrics used in this analysis were (1) the Pearson *correlation* between cells’ rank order along the inferred trajectory and their rank order along the true trajectory and (2) the proportion of cells for which the inferred branch *overlapped* (i.e., was consistent) with the branch in the true trajectory. Both of these metrics have been previously described and used to evaluate inferred cell trajectories in [18].

**Fig. 5:**
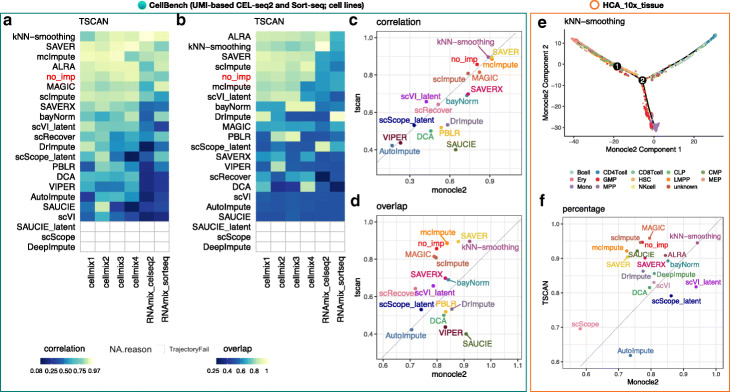
Impact of imputation methods on inferred trajectories for pseudotime analysis. **a** Heatmap showing the Pearson correlation coefficients (PCC), denoted as *correlation*, between the ranks of the inferred trajectory using TSCAN [54] and the rank order of the cells where we know the true trajectory (or ordering) of the cells, using the six RNA mixture and cell mixture datasets from CellBench [18]. White areas with gray outline in **a** and **b** indicate that TSCAN failed to infer trajectories from the imputed profiles. **b** Heatmap of the proportion of cells on the inferred trajectories from TSCAN that correctly *overlap* with the cells on the branch where we know the true trajectory of the cells using the same data as **a**. **c** The comparison of the correlation and **d** overlap averaged across datasets using Monocle 2 [53] (*x*-axis) and TSCAN [54] (*y*-axis) as the trajectory reconstruction method. **e** An inferred trajectory from Monocle 2 using *N*=6941 bone marrow cells from the *HCA_10x_tissue* that were imputed using kNN-smoothing [27]. Colors represent computationally defined cell types using bulk RNA-seq data (see the “Methods” section for details). **f** Here, we compare the estimated pseudotime to the level of differentiation for each pair of bone marrow cells. For instance, for the pair of cells A and B, if cell A is a hematopoietic stem cell (HSC) (we assign it as differentiation level 1), cell B is a multipotent progenitor (MPP) cell (we assign it as differentiation level 2), and the inferred pseudotime for cells A and B is *t*_*A*_ and *t*_*B*_ where *t*_*A*_<*t*_*B*_, then we call it “correctly ordered.” The *percentage* of the correctly ordered cells averaged across all possible cell pairs serves as the assessment measure considering both Monocle 2 (*x*-axis) and TSCAN (*y*-axis)

Using the CellBench data, we found the imputation methods kNN-smoothing, SAVER, and ALRA led to both increased correlation (Fig. 5a) and overlap (Fig. 5b) compared to no imputation using the TSCAN trajectory inference. Using Monocle 2 trajectory inference, SAVER, kNN-smoothing, mcImpute, and the latent spaces from SAUCE (SAUCE_latent) increased both the correlation (Additional file 1: Figure S10a) and overlap (Additional file 1: Figure S10b) compared to no imputation. This confirms the variability in imputation methods’ performance (depending on trajectory inference method), in particular for the overlap, that was previously shown [18] (Fig. 5c–d). Finally, analogous to results shown in clustering evaluation, for imputation methods that return latent spaces, using the latent spaces generally led to better performance than using the imputed expression values.

Next, we evaluated the performance of the imputation methods using bone marrow cells from the *HCA_10x_tissue* [11]. Because these are not cell lines, we first computationally labeled cell types using bulk RNA-seq data (see the “Methods” section for details). We used the schematic of hematopoietic stem cell (HSC) differentiation shown in Fig. 1a in Buenrostro et al. [55] to computationally label cell types. The bone marrow contains hematopoietic stem cells (HSCs) differentiating into three major lineages: lymphoid, erythroid, and myeloid cells (Fig. 5e). Here, we compare the estimated pseudotime to the level of differentiation for each pair of bone marrow cells. For instance, for the pair of cells A and B, if cell A is a HSC cell (we assign it as differentiation level 1), cell B is a multipotent progenitor (MPP) cell (we assign it as differentiation level 2), and the inferred pseudotime for cell A and B is *t*_*A*_ and *t*_*B*_ where *t*_*A*_<*t*_*B*_, then we call it “correctly ordered.” The *percentage* of the correctly ordered cells averaged across all possible cell pairs serves as the performance metric.

Similar to using the CellBench data, kNN-smoothing most consistently outperformed other imputation methods using this heterogeneous tissue data using either TSCAN or Monocle 2. However, there was variability in the performance of other imputation methods depending on the trajectory analysis method used (Fig. 5f), as previously reported [18]. Figure 5f shows the percentage of correctly ordered cell pairs in the bone marrow dataset using Monocle 2 (*x*-axis) and TSCAN (*y*-axis) as the pseudotime reconstruction methods. We found kNN-smoothing and MAGIC slightly performed better than using no imputation. However, the majority (all but two) of methods reported 78%+ correctly ordered cells in this data using either TSCAN or Monocle 2.

## Discussion

We have presented a systematic benchmark evaluation comprehensively comparing 18 scRNA-seq imputation methods. Our comparison is subject to several limitations. Firstly, the imputation methods were mostly compared with default parameters which may not achieve optimal performance across all datasets. Our work could be further improved with the use of methods such as molecular cross-validation (MCV) [56]. In addition, we used 72 h as the time limit for convergence for imputation methods, which does not guarantee algorithmic convergence for some methods. In our evaluation of imputation methods on inferring pseudotime with trajectory analysis methods, the cell types of the tissue *HCA_10x_tissue* cells were computationally annotated. Another limitation is that there are no disease tissues included in this study. In the future, it is worthwhile to continue investigating how conclusions presented in this study may translate to applications in a diseased setting such as cancer tissues. One challenge is that the expression of some genes in diseased cells might be abnormal [57–60], which could lead to the false identification of similar cells and could affect the imputation performance.

An open problem not investigated in our current study is the impact of imputation methods on the RNA velocity analysis [61–63]. Since RNA velocity is estimated by analyzing unspliced and spliced mRNA, it takes into account both spliced and unspliced counts. Existing imputation methods deal with the drop-out events by imputing the gene expression values rather than the original reads, and the gene expression is usually quantified on exons only which may not distinguish contributions from the spliced versus unspliced transcripts. Therefore, whether existing imputation methods can also be applied to velocity analysis and to separately impute spliced and unspliced transcripts (including introns) remains an open problem that requires extensive future investigation which is beyond the scope of the present study. In addition to the velocity analysis, evaluating how imputation methods may impact other emerging analyses such as spatial transcriptomics [64–68] also warrants future investigation.

## Conclusions

**Fig. 6:**
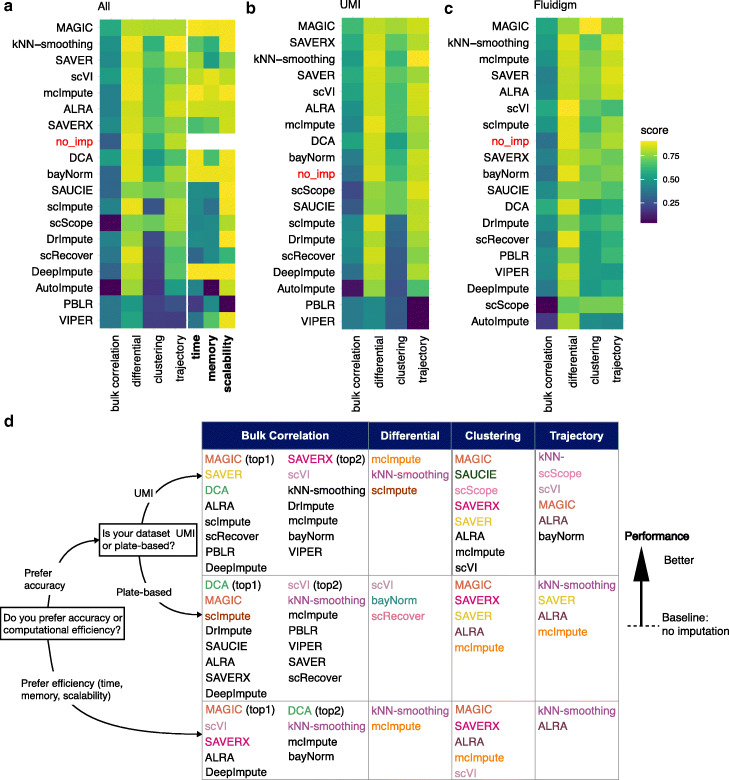
Overall summary of results evaluating imputation methods for scRNA-seq data. Performance of imputation methods in all evaluation aspects: similarity between imputed single-cell and bulk profiles (denoted as “bulk correlation”), differential expression (“differential”), unsupervised clustering (“clustering”), trajectory inference (“trajectory”), time, memory usage, and scalability. The units of computational time (in minutes), memory usage (in maximum resident set size of all tasks in job (MaxRSS or maximum resident set size of all tasks in a job) in gigabytes (GB)), and scalability (w.r.t. the number of observations or cells) were all scaled to be in [0,1] to apply the same color scale. For more details on units of computational time, memory usage and scalability, see the “Methods” section and details in Additional file 1: Figure S11. A higher score represents a better performance. Imputation methods are ranked by averaging the scores in bulk correlation, differential, clustering, and trajectory. No imputation is abbreviated as “no_imp”. **a** Performance scores and the ranking of all imputation methods using datasets across UMI-based and plate-based (Fluidigm) protocols; **b** using UMI-based data only; **c** using Fluidigm data only. **d** Practical guidelines for method users. For users who prefer imputation accuracy (UMI-based and plate-based rows in the table), we listed methods that perform better than no imputation for each analysis task. For each task, methods with better performance are listed on top, and the top five methods are displayed in color. For users who prefer computational efficiency (last row in the table), we first obtained methods that were ranked in top 50% in terms of overall time, memory, and scalability performance. Then, for each analysis task, we retained methods that outperformed no imputation in terms of accuracy, and we listed these methods in the table in the rank order based on their overall accuracy performance (averaged across UMI- and plate-based platforms) in each analysis

A good imputation method should allow one to accurately recover gene expression. Ideally, it should improve downstream analyses without introducing many artifacts or false signals. Motivated by this, we evaluated the performance of the imputation methods based on the similarity between imputed single-cell profiles and bulk profiles in a homogeneous population of cells, and the impact of the imputation methods on three downstream analyses: differential expression analysis, unsupervised clustering analysis, and pseudotime inference. We conclude by summarizing our results in Fig. 6 which ranked methods based on their average performance. Computational time, memory usage, and scalability were not used to rank the methods. However, they were assessed separately using four datasets of 10^3^,5×10^3^,5×10^4^ and 10^5^ cells, respectively. Specifically, we used (1) computation time (in seconds), (2) memory (in maximum resident set size of all tasks in job, i.e., MaxRSS, returned from sacct), and (3) scalability—regression coefficient in the linear model where the computation time is fitted against the number of cells on the log10-scale. The Additional file 1: Figure S11 shows the comparison of time, memory, and scalability. As a method’s performance depends on the analysis task and the experimental platform used to generate the data, Fig. 6a–c provides heatmaps that summarize the performance of different methods in different analysis tasks and for different experimental platforms. Figure 6d also summarizes the methods with performance better than no imputation (methods shown in ranked order) for each analysis task and data platform. It can serve as a map to guide users and investigators to navigate the current space of scRNA-seq imputation methods depending on their needs. In addition, since the input data requirements and processing procedures are variable among methods, we also outlined these details in Additional file 5: Table S4 for user’s reference.

Of the methods considered, MAGIC, kNN-smoothing,and SAVER were found to outperform the other methods most consistently (Fig. 6). However, the performance of methods varied across evaluation criteria, experimental protocols, datasets, and downstream analyses. For example, scVI was one of the top performers in terms of the similarity between the imputed single-cell and bulk expression profiles (Fig. 2), but it did not perform among the top in clustering and trajectory analysis (Figs. 4 and 5). SAVER-X performed well in UMI-based data, but less well in non-UMI based data (Fig. 6). While MAGIC was one of the top performers overall (Fig. 6), it performed worse than many other methods when identifying differentially expressed genes in hypothesis testing type settings that take into account cell variability (Fig. 3).

In addition, we found that while some imputation methods improve detecting differentially expressed genes or discovering marker genes, they also can introduce false positive signals, sometimes driven by imbalanced cell numbers between groups (e.g., Additional file 1: Figure S2i-j). The magnitude (i.e., effect size) of differential expression (i.e., log-fold change) plays a role in the performance of the imputation methods. Most imputation methods strengthen large effect sizes compared to no imputation. However, if the original expression difference is small, then most imputation methods may smooth away the small differential signal and hence do not show clear advantage over not imputing (Fig. 3j, k).

One important observation is that, while the majority of imputation methods outperformed no imputation in recovering bulk expression (16 out of 18 methods) and log fold changes of individual genes between cell types without considering cell variability within each cell type (13 out of 18 methods) (Fig. 2), much fewer methods performed better than no imputation for identifying differentially expressed genes after considering cell variability (1–12 out of 18 depending on the test scenario), clustering cells (6–10 out of 21 methods) or inferring pseudotemporal trajectories (4–11 out of 21 methods) (the “Results” section). Thus, the current imputation methods as a whole seem to be most effective for providing a point estimate of the activity of individual genes, and they become less effective when coupled with various downstream analysis tasks. For differential expression analysis, the decreased effectiveness is likely due to inaccurate cell variance characterization after imputation. For clustering and trajectory analysis, the reduced effectiveness is likely because these two analyses attempt to analyze cell-to-cell relationship rather than individual genes. Cell clustering and trajectory analysis are usually conducted by embedding the high-dimensional expression vector of each cell into a relatively low-dimensional space. Each dimension in the low-dimensional space combines information from many genes, which increases signal-to-noise ratio by diluting technical noise such as observed zeros due to technical variation, even without imputation. Thus, the recovery of cell-to-cell relationship can be influenced less by imputation. By contrast, the measurements of individual genes contain high level of technical noises which can be greatly mitigated by imputation by borrowing information from other genes or cells. Thus, imputation could be more helpful for analyzing individual genes rather than cell-to-cell relationship. An open question to be investigated in the future is whether the improvement on the various downstream analysis tasks by imputation has already reached its upper limit and, if not, how to design new imputation methods to further improve the analysis of cell-to-cell relationship or differential expression that takes into account cell variability.

In terms of computation, MAGIC, DCA, and scVI are among the most efficient methods according to the average of three metrics: time, memory, and scalability. SAUCIE, scScope, DeepImpute, ALRA, and kNN-smoothing exhibit high scalability with increasing numbers of cells in datasets. scImpute and bayNorm are intermediary while the remaining methods do not scale well for large datasets (Additional file 1: Figure S11).

Our systematic benchmark evaluation highlights the advantages and disadvantages of existing imputation methods and that the performance of an imputation method depends on many external factors, such as experimental protocols and analyses usage. We hope that this study can benefit both users and method developers and provide an evaluation standard for future scRNA-seq imputation methods.

## Methods

All methods were evaluated with default parameters, with the exception of the deep-learning-based methods for which the maximum epoch time was set as 400. We used 72 h as the time limit for convergence for the imputation methods. This did not guarantee algorithmic convergence for some methods. For a description of the data, see the “Data” section and Additional file 4: Table S3. For complete details on the methods used, the input, the output, pre-processing steps, the programming language used, version number, and link to software, see Additional file 5: Table S4.

### Overview of scRNA-seq imputation methods

The 18 imputation methods are reviewed and summarized in Additional file 5: Table S4. We grouped them into four categories.

1. *Model-based methods:* These include 6 methods, namely, bayNorm [19], SAVER [20], SAVER-X [21], scImpute [22], scRecover [23], and VIPER [24]. These methods assume that the data follow a specific model. SAVER models the UMI counts using a negative binomial distribution. SAVER-X is similar to SAVER but has an additional option to pretrain hyperparameters using existing datasets. scRecover is based on a zero-inflated negative binomial distribution model which tries to adapt to high drop-out rates. bayNorm uses a binomial model of mRNA capture for its likelihood function. scImpute assumes the dropout rate of each gene follows a double exponential function. VIPER is based on a sparse nonnegative regression model.
2. *Smoothing-based methods:* These include 3 methods, namely, DrImpute [25], MAGIC [26], and kNN-smoothing [27]. DrImpute first clusters cells and then averages the expression values from similar cells. MAGIC performs data diffusion on the Markov affinity-based matrix for the imputation of cells. In contrast, kNN-smoothing models technical variance using a Poisson distribution and based on that, kNN-smoothing smooths each cell by its *k*-nearest neighbors.
3. *Deep learning methods:* These include 6 methods, namely, AutoImpute [28], DCA [29], DeepImpute [30], SAUCIE [31], scScope [32], and scVI [33]. In these methods, a latent space is constructed using deep learning models to represent cells by low-dimensional latent variables which are used to reconstruct gene expression. The latent space representation can be used for downstream analyses, such as clustering the cells or inferring pseudotime trajectories on the cells, but not for differential gene expression analysis. DCA is a deep count autoencoder network that uses a negative binomial noise model with or without zero-inflation (depending on the dispersion learned form data) and captures nonlinear gene-gene dependencies. scVI is based on a hierarchical Bayesian model and applies deep neural networks to specify the conditional distributions of variables where the latent variables are mapped to the zero-inflated negative binomial distribution. AutoImpute applies overcomplete autoencoders and tends to be more conservative by considering the expression values as truly zeros if the genes are silenced across bulk samples. DeepImpute constructs multiple sub-neural networks to impute sets of target genes using genes highly correlated with the target genes. SAUCIE is a regularized autoencoder that uses the reconstructed signal from autoencoder to denoise and impute the data. ScScope iteratively performs imputation using a recurrent network layer.
4. *Low-rank matrix representation methods:* These include 3 methods, namely, ALRA [34], mcImpute [35], and PBLR [36]. In these low-rank matrix-based methods, cell profiles are mapped to a low-dimensional linear space for imputation. ALRA uses SVD decomposition followed by a thresholding scheme. mcImpute uses nuclear norm minimization, a matrix completion algorithm. PBLR first groups cells into subpopulations and then runs a bounded low-rank matrix recovery method within each cell subpopulation.

Note that both deep-learning-based methods and low-rank-matrix-based methods use the idea of data reconstruction. Regarding the programming languages, the deep-learning-based methods were all implemented using Python. mcImpute and PBLM were implemented using MATLAB. MAGIC and SAVER-X are available in both R and Python. All others are implemented using R. For more details about the methods, see Additional file 5: Table S4.

### Data

#### Single cells and bulk samples from cell lines

1. Single cells and pseudo cells from the CellBench [18] scRNA-seq benchmarking dataset
  All UMI-based data from five cell lines (HCC827, H1975, H2228, H838, A549) in the CellBench [18] benchmarking dataset (except for *cellmix5*, a population control) using the CEL-seq2 protocol (*sc_celseq2*, *sc_celseq2_5cl_p1*, *sc_celseq2_5cl_p2*, *sc_celseq2_5cl_p3*, *cellmix1*, *cellmix2*, *cellmix3*, *cellmix4*, *RNAmix_celseq2*), Drop-seq Dolomite protocol (*sc_dropseq*), the Sort-seq protocol (*RNAmix_sortseq*), and 10x Chromium Genomics protocol (*sc_10x*, *sc_10x_5cl*). For a description of the experimental design, GEO accession numbers, protocol parameters, see thesc_mixologyGitHubrepo and Additional file 4: Table S3.
  *N*=10 bulk RNA-seq samples from GSE86337[69] (each of the five cell lines have two replicates)
2. Five ENCODE (*A549*, *GM12878*, *h1-hESC*, *IMR90*, *K562*) cell lines
  *ENCODE_fluidigm_5cl*: The five cell lines correspond to five cell types and contain a total of *N* = 362 cells all from GSE81861. They were sequenced with the SMARTer full-length method [17] using the Fluidigm C1 protocol [70]. The cell types include *N* = 74 A549 cells, *N* = 96 GM12878 cells (batch 2), *N* = 96 H1-hESC cells (batch 1) referred to as *H1* in the manuscript, *N* = 23 IMR90 cells, and *N* = 73 K562 cells.
  The bulk RNA-seq samples from ENCODE [71]: *N* = 7 A549 samples, *N*= 11 GM12878 samples, *N* = 8 H1-hESC samples, *N* = 5 IMR90 samples, and *N* = 27 K562 samples
3. *Jurkat* cell lines
  *10x_293t_jurkat* (293T cells): *N* = 3258 cells measured using UMIs and the droplet-based protocol from 10x Genomics [3] (https://support.10xgenomics.com/single-cell-gene-expression/ datasets/1.1.0/jurkat)
  *N* = 2 bulk RNA-seq sample (GSE129240[72])
4. *HEK293T* cell lines
  *10x_293t_jurkat* (jurkat cells): *N* = 2885 cells measured using UMIs and the droplet-based protocol from 10x Genomics [3] (https://support.10xgenomics.com/single-cell-gene-expression/ datasets/1.1.0/293t)
  *N* = 2 bulk RNA-seq sample (GSE129240[72])

#### Single cells and bulk samples from tissues

1. Bone marrow tissue from the Human Cell Atlas [11] (HCA)
  *HCA_10x_tissue*: *N* = 6939 bone marrow cells from sample MantonBM6 measured using 10x Genomics (https://data.humancellatlas.org/explore/projects/ cc95ff89-2e68-4a08-a234-480eca21ce79)
  *N*= 49 bulk RNA-seq samples from 13 cell types (B cell, CD4 T cell, CD8 T cell, CMP, GMP, HSC, MEP, Monocyte, MPP, and NK cells each has 4 samples and each of CLP, Erythroid and LMPP has 3 samples). (GSE74246[73])
2. Sorted peripheral blood mononuclear cell (PBMC) tissue from 10x Genomics (UMI)
  *PBMC_10x_tissue*: *N*= 59620 sorted cells from 10 cell types (*N* = 4033 B cells, *N* = 498 CD14 monocyte cells, *N* = 9162 CD34 cells, *N*= 7046 CD4 T helper cells, *N* = 7555 CD56 NK cells, *N*= 7631 cytotoxic T cells, *N* = 6969 memory T cells, *N* = 5672 naïve cytotoxic cells, *N* = 4569 naïve T cells, and *N* = 6485 regulatory T cells) (http://support.10xgenomics.com/single-cell/datasets).

### Data processing and imputation

#### Single-cell RNA-seq data

We applied the same quality control (QC) criterion across all single-cell datasets. Cells with at least 500 detected genes were retained. ERCC spike-ins were removed. Genes expressed in at least 10% of cells in cell line data and 1% of cells in tissue samples were retained. Mitochondrial genes were removed. We applied these cell- and gene-filtering steps to all imputation methods and skipped each method’s own gene- and cell-filtering (if applicable) to keep the dimension of the imputed values (output) the same across imputation methods. Single-cell profiles from five Fluidigm-based ENCODE cell lines (*ENCODE_fluidigm_5cl*) were combined into one count matrix which was used as input to each imputation method. A similar procedure was applied to other datasets. Single-cell profiles from UMI-based *Jurkat* and *HEK293T* cell lines were also combined into one UMI count matrix (*10x_293t_jurkat*) which was used as input to each imputation method. Data were normalized by the pooling normalization method [37] implemented in the scran [38] R/Bioconductor [39, 40] package and log2-transformed if any imputation method requires normalized counts or log-transformed normalized counts as input. We added post-processing steps for methods if required. For example, if a method did not normalize nor apply a log-transformation before or during imputation, we applied scran normalization and log2-transformation to the output. If a method included normalization but does not log-transform the data during imputation, we applied log2-transformation to the output.

#### Bulk RNA-seq data

Read counts were downloaded from GEO (accession numbers GSE129240, GSE86337, and GSE74246). Each sample was normalized by library size and multiplied by a size factor 10^6^, or count per million (CPM), and then log2-transformed with a pseudocount of 1, i.e., log2(*CPM*+1). For the ENCODE samples, the downloaded data were already normalized (Fragments Per Kilobase of transcript per Million mapped reads, or FPKM) and log2-transformed.

### Null simulation

To generate Fig. 1a and Additional file 1: Figure S1, we used the 293T cells from the *10x_293t_jurkat* dataset with *G*=18729 genes and *C*=2885 cells. We applied gene-level quality control by only retaining genes that have expression at least 10% of cells, which kept *G*=7582 genes. Let *X*_*gi*_ represent the observed scRNA-seq UMI counts for the *g*^*th*^ gene where *g*∈(1,…,*G*) and the *i*^*th*^ cell where *i*∈(1,…,*C*) from a given dataset. We estimated the mean expression level for each gene across the cells: $\hat {\mu }_{g} = \sum _{i=1}^{C} \frac {X_{gi}}{C}$. Then, for each gene, we simulated scRNA-seq UMI counts *Y*_*gj*_ for the *g*^*th*^ gene and the *j*^*th*^ simulated cell from a Poisson distribution with the mean equal to the product of the estimated gene-level mean ($\hat {\mu }_{g}$) estimated from **X** and a cell-specific library size factor (*δ*_*j*_) randomly sampled from a uniform distribution between [*a*,*b*]:

$$Y_{gj} \sim Pois(\mu_{g} \times \delta_{j})$$

where *δ*_*j*_∼*U*[*a*,*b*] for *j*∈(1,…,*C*^′^). For Fig. 1a and Additional file 1: Figure S1, *C*^′^ = 1000 and the library size factors *δ*_*j*_∼*U*[0.9,1.1]. We passed the gene expression matrix to each imputation method. Data preprocessing and postprocessing steps followed the “Data processing and imputation” section. Principal component analysis was performed on genes with coefficient of variation (cv) greater than the median cv.

### Evaluation of similarity between bulk and imputed scRNA-seq data

#### Correlation of gene expression profiles between bulk and imputed scRNA-seq profiles

For a given scRNA-seq dataset, we averaged the scran normalized [37] log2-transformed scRNA-seq counts across cells (referred to as a “pseudobulk”) and calculated the Spearman’s rank correlation coefficient (SCC) (or *ρ*) between the pseudobulk and the bulk RNA-seq profile (averaged across replicate bulk samples) of the same cell type. Next, for each cell in a given scRNA-seq dataset, we calculated the SCC between the cell’s imputed scRNA-seq profile (e.g., using SAVER) and the averaged bulk RNA-seq profile. The median SCC across all cells was then used to evaluate the performance of an imputation method within a dataset. To rank methods across datasets, we first averaged the median SCCs across datasets within the same experimental protocol (e.g., UMI-based or plate-based) and then averaged these averages across protocol. In the analyses above, pseudobulk was used as a reference for the approximate upper bound of the single-cell imputation performance since both bulk and pseudobulk profiles try to measure a cell population’s average behavior, and the correlation between a pseudobulk and the corresponding bulk profile is expected to increase as one pools an increasing number of cells to create the pseudobulk sample [74, 75]. Note that unlike single-cell profiles, pseudobulk cannot characterize cell-to-cell variability. Thus, pseudobulk cannot replace single-cell data in studies that require analyzing cell-to-cell variability, such as a rigorous differential expression analysis that aims to detect differences between two cell types that cannot be explained by the background cell-to-cell variation.

To see whether there exist significant platform differences, two-sample *t*-test was conducted to evaluate whether the Spearman’s correlation coefficients (SCC) between the bulk and imputed single-cell data for each method had equal mean or different means across different platforms (e.g., 10x vs. Fluidigm). The *p* values from all methods were then adjusted for multiple testing using the Benjamini-Hochberg false discovery rate (FDR) method [76]. Methods with FDR < 0.05 and relative performance change > 25*%* were highlighted in Fig. 2d, h. Here, relative performance change is defined as $ |\overline {SCC}_{10x}-\overline {SCC}_{fluidigm}| / max(\overline {SCC}_{10x},\overline {SCC}_{fluidigm})$, where $\overline {SCC}$ represents mean imputed value-bulk SCC across all datasets within a platform.

To quantify the relationship between the number of cells in a dataset and the imputation performance, we calculated the Spearman correlation between the cell number and a method’s imputation performance (i.e., SCC between the bulk and imputed single-cell data) across datasets for each imputation method within each platform (e.g., 10x or Fluidigm).

#### Correlation between the bulk and imputed single-cell gene expression log fold changes

This analysis was similar to the above one, but here the SCC was calculated by comparing bulk and single-cell differential expression (DE). For a given pair of cell types, we first averaged the cell profiles from each cell type to form two “pseudobulk” samples using scran normalized [37] and log2-transformed scRNA-seq profiles. The difference between the two pseudobulk profiles and the difference between the two bulk RNA-seq profiles were computed and the SCC between the two differential profiles was computed.

Next, we took one cell from each cell type and calculated the difference in the imputed scRNA-seq profile (e.g., using SAVER) between these two cells. The SCC between this difference and the difference between two bulk RNA-seq profiles from the same two cell types was then computed. This was repeated for all possible cell pairs. For a given pair of cell types (both within the same experimental protocol), the median SCC across all cell pairs was computed. To rank methods across datasets, we averaged the median SCCs across datasets within each protocol and then averaged these averages across protocols.

### Evaluation of imputation to identify differentially expressed genes

#### Ranking differentially expressed genes (DEGs)

For each of the three imputed scRNA-seq datasets (*sc_10x_5cl*, *ENCODE_fluidigm_5cl* and *HCA_10x_tissue*), we identified differentially expressed genes (DEGs) between all pairs of cell types. We considered two methods to identify DEGs from scRNA-seq: (1) MAST [46] which models the data using a hurdle model and (2) Wilcoxon rank-sum test [47]. For bulk RNA-seq samples, DEGs were identified using the limma [77] R/Bioconductor package. We corrected *p* values for multiple testing using the Benjamini-Hochberg (BH) method [76] (p.adjust function in the stats R package) to derive false discovery rate (FDR) (only when a method did not already correct for FDR) and identified genes with FDR smaller than *α*=0.05. The bulk DEGs were treated as a “gold standard” similar to previous studies [48]. For the scRNA-seq data, The single-cell DEGs were ranked by *p* values or the log-scaled expression fold change if there was a tie for *p* values. For *i* from 1 to 100, we calculated the proportion of top 10∗*i* single-cell DEGs that overlap with bulk DEGs. The average of these 100 proportions served as the performance metric.

#### Null differential analysis

For each dataset in this analysis, we started with a homogeneous population of cells where we expect no DEGs after correction for multiple testing. We used the A549 cells from the *sc_10x_5cl* dataset (*N*=1256 cells), the GM12878 cell line from the *ENCODE_fluidigm_5cl* dataset (*N*=96 cells), and the cell type with the largest number of cells from the *HCA_10x_tissue* (*N*=193 monocytes). For each dataset, we randomly sampled cells into two groups with group size ranging from *N* = 10 to 500 cells per group, imputed the expression values of these two groups together and identified DEGs using MAST [46] and Wilcoxon rank-sum test [47]. The genes with FDR < 0.05 were identified as DEGs.

#### Predicting cell type using imputed expression of known PBMC marker genes

Using the sorted peripheral blood mononuclear cells (PBMCs) [3] (*PBMC_10x_tissue* dataset), we assessed the performance of an imputation method on recovering the expression level of known PBMC marker genes. The cell type-specific marker genes used in this analysis were the following: CD19 for B cells; CD14 for monocytes; CD34 for CD34 + cells; CD3D for CD4 T helper cells, cytotoxic T cells, memory T cells, naive cytotoxic T cells, naive T cells, regulatory T cells; CD4 for CD4 T_helper cells, memory T cells, naive T cells, regulatory T cells; CD8A for cytotoxic T cells and naive cytotoxic T cells. We evaluated the performance of predicting a cell type (e.g., B cell) based on the imputed expression of a marker gene (e.g., CD19). For each cell type and marker gene pair, we calculated the area under the receiver operating characteristic (ROC) curve (AUROC) using the performance function in the ROCR R package[78] where the expression of the marker gene is the predictor and the true cell type is the label. Specifically, the cells were first sorted in a descending order according to the imputed values of the marker gene (e.g., CD19). Consider the cell type *A*. Assume there were *K* cells in total and *b* of them were in cell type *A*. Assume in the top *N* cells, *a* of them were in cell type *A*; in the remaining *K*−*N* cells, *c* of them were not in cell type *A*. Sensitivity was calculated as *a*/*b* and specificity was calculated as *c*/(*K*−*b*). The ROC curve was obtained by plotting sensitivity against 1-specificity for different *N* (*N*=1,2,...,*K*).

#### Types of failures

In the differential expression analysis, there are three types of failures.

1. “ImputationFail”: It means no imputation results were returned after running the imputation methods for 72 h. Without imputed values, one cannot proceed with the differential expression analysis. This type of failure is due to failure of the imputation methods, so we assign a zero score to the method, and the score will be counted towards the method’s overall performance in the evaluation and affect the ranking of the imputation methods.
2. “DifferentialFail”: Here, the imputation methods can impute the gene expression, but the methods for detecting differentially expressed genes failed to run. In this scenario, imputation methods themselves did not fail, but one does not have enough information to compare their imputation performance since differential expression analysis cannot be run. Therefore, this type of failure will not be counted in evaluation, and the test results provided by the other test method will be counted only (in our analysis, Wilcoxon rank-sum test can always provide test results).
3. False positives and false negatives: Here, both the imputation methods and the differential expression analysis methods (e.g., MAST) can be run without encountering technical issues. However, one may incorrectly identify differentially expressed genes from the imputed data. This further includes two scenarios: (a) false positives: a non-differential gene is incorrectly reported as differential; (b) false negatives: a differential gene is incorrectly reported as non-differential. Null differential analysis (Fig. 3e–h) specifically evaluates false positives by asking each method to detect differential genes in null analyses where no differential genes are expected. The comparisons to ranking differentially expressed genes (Fig. 3a–d), on the other hand, evaluates each method’s ability to put truly differential genes to top rank which depends on both false positives and false negatives. For a given rank (say top *N* genes), a better method should have fewer false positives among the top *N* reported genes and fewer false negatives in the remaining genes. This type of failure will be considered in the evaluation of imputation methods’ overall performance.

### Evaluation of imputation on unsupervised clustering

We used two sets of datasets for this analysis. The first set is CellBench [18] data, which consists of 7 datasets. Three of these datasets contained three cell lines (datasets: *sc_10x*, *sc_dropseq*, *sc_celseq2*) and four datasets contained five cell lines (datasets: *sc_10x_5cl*, *sc_celseq2_5cl_p1*, *sc_celseq2_5cl_p2*, *sc_celseq2_5cl_p3*). The second set of data contains sorted PBMCs [3] (*N* = 59620) with 10 cell types.

Clustering was performed using both *k*-means [49] and Louvain clustering [50] where the number of clusters was set to be the number of known cell types known in each dataset. *K*-means clustering was performed using the top 10 PCs. In *k*-means, we directly set *k* to be the number of known cell types, while in Louvain clustering this is achieved by increasing the number of nearest neighbors iteratively until the desired number of clusters is obtained. Louvain clustering was performed by performing feature selection using highly variable genes (HVGs), applying PCA by prcomp(), and then building a shared *k*-nearest-neighbors (*k*NN) graph [50] based on the Euclidean distances of the top 10 PCs. An edge was drawn between all pairs of cells sharing at least one neighbor, weighted by the characteristics of the shared nearest neighbors. For this last step, we used the buildSNNGraph function in the scran [38] R/Bioconductor package with the top 10 PCs as input, *d*=*NA* and other parameters as default. Clusters were identified by a multi-level modularity optimization algorithm for finding community structure [79] using cluster_louvain in igraph R package[80]. For scVI_latent, scScope_latent, and SAUCIE_latent, we skipped the principal component analysis of the imputed values and replaced the top 10 PCs using the latent space coordinates obtained from scVI, scScope, and SAUCIE.

To evaluate the performance of each method, we used four metrics:

1. Entropy of accuracy (*H*_*acc*_) where 0≤*H*_*acc*_≤ log*M*. *H*_*acc*_ measures the diversity of the ground-truth labels within each predicted cluster group assigned by the clustering algorithm.
  $$H_{acc} = - \frac{\sum_{i=1}^{M} \sum_{j=1}^{N_{i}} p_{i}(x_{j}) \log(p_{i}(x_{j}))} {M} $$
  where *M* is the total number of predicted clusters from the clustering algorithm, *N* is the number of ground-truth clusters, and *N*_*i*_ is the number of ground-truth clusters in the *i*^*th*^ predicted cluster. *x*_*j*_ are cells in the *j*^*th*^ ground-truth cluster, and *p*_*i*_(*x*_*j*_) are the proportions of cells in the *j*^*th*^ ground-truth cluster relative to the total number of cells in the *i*^*th*^ predicted cluster. A smaller value of *H*_*acc*_ is better as it means the cells in a predicted cluster are homogeneous and from the same group [18]. However, *H*_*acc*_ can lead to over-clustering, with an extreme case being treating each cell as a cluster (or *H*_*acc*_ = 0).
2. Entropy of purity (*H*_*pur*_) where 0≤*H*_*pur*_≤ log*N*. *H*_*pur*_ measures the diversity of the predicted cluster labels within each of the ground-truth groups.
  $$H_{pur} = - \frac{\sum_{i = 1}^{N} \sum_{j = 1}^{M_{i}} p_{i}(x_{j}) \log(p_{i}(x_{j}))}{N} $$
  where *N* is the total number of ground-truth clusters, *M*_*i*_ is the number of predicted clusters in the *i*^*th*^ true cluster. *x*_*j*_ are cells in the *j*^*th*^ predicted cluster, and *p*_*i*_(*x*_*j*_) are the proportions of cells in the *j*^*th*^ predicted cluster relative to the total number of cells in the *i*^*th*^ ground-truth cluster. A smaller value of *H*_*pur*_ is better as it means the cells in the ground-truth groups are homogeneous with the same predicted cluster labels [18]. However *H*_*pur*_ can lead to under-clustering, with an extreme case being assigning all cells into one predicted cluster so that each of the ground-truth groups has the same predicted cluster label (*H*_*pur*_ = 0).
3. Adjusted Rand index [51] (ARI). We used the adjustedRandIndex function in mclust package [81]. The minimum ARI of each dataset was obtained by permuting cells’ ground-truth cell type labels 10^4^ times, recomputing ARI, and averaging the 10^4^ ARIs from random permutations. The maximum has been theoretically proved as 1. We subtract the ARI by the empirical minimum and divided by the distance between the empirical minimum and theoretical maximum.
4. Median Silhouette index *S*=*Median*(*s*(*i*)) where *i* is a cell, *C*_*i*_ is the set of cells in the same cluster as *i*, |*C*_*i*_| is its cardinality (i.e., number of cells in a cluster),
  $$s(i) = \begin{cases} 1 - a(i) / b(i) & \text{if} a(i) < b(i) \\ 0 & \text{if} a(i) = b(i) \\ b(i) / a(i) -1 & \text{if} a(i) > b(i) \end{cases} $$
  and $a(i) = \frac {1}{|C_{i}| -1} \sum _{k \in C_{i}, k\neq i} d(i,k), b(i) = \min _{i\neq j} \frac {1}{|C_{j}|} \sum _{k\in C_{j}} d(i,k)$. Here *d*(*i*,*k*) denotes the Euclidean distance between cells *i* and *k*. This is implemented by silhouette function in the cluster R package [82]. The range of S is [−1,1].

### Identify cell types in *HCA_10x_tissue* dataset

To computationally identify the cell types in the *HCA_10x_tissue* dataset, we downloaded bulk RNA-seq profiles from 13 primary hematopoietic cell types (GEO accession number GSE74246). The data were normalized and scaled using log2-transformed TPM. We averaged bulk profile replicates from the same cell type. We used the bulk profiles to computationally assign a cell type label for each individual cell. Consider the *j*^*th*^ bulk cell type profile. We calculated the log-fold change (LFC) between the *j*^*th*^ bulk cell type profile and each of the other bulk profile and identified the top 100 genes with the largest LFC for each comparison, which were used to define anchor gene sets. Repeating this for all bulk cell types resulted in 13×12=156 anchor gene sets. Next, for each bulk cell type or each single cell, we calculated mean expression of anchor genes in each of these gene sets, yielding a vector of length 156 which we refer to as “anchor profile” of a cell type or a cell. We calculated the Spearman correlation between each single cell anchor profile and each bulk cell type anchor profile. A cell was computationally assigned to the *j*^*th*^ cell type if the bulk profile of this cell type had the largest correlation with the single cell and the correlation coefficient was greater than 0.6. If no cell type label was assigned to a cell in this way, the cell’s cell type was labeled as unknown. Next, *k*-means clustering was performed on the cells. For each cluster, if at least 70% of cells were inferred as cell type *A*, then all cells in the cluster were relabeled as cell type *A*. If a cluster cannot be labeled in this way, then all cells in the cluster were labeled as unknown cell type. The largest cluster obtained in this procedure contained *N* = 193 cells inferred as monocytes. They were used for the null differential analysis described before.

### Evaluation on pseudotime inference

#### Constructing trajectories

We used the (1) CellBench [18] RNA mixture (*RNAmix_celseq2*, *RNAmix_sortseq*) and cell mixture (*cellmix1*, *cellmix2*, *cellmix3*, *cellmix4*) datasets from three cell lines (H2228, H1975, HCC827) generated by CEL-seq2 and SORT-seq protocols [18] and (2) bone marrow cells from the HCA_10x_tissue [11]. Monocle 2 [53] and TSCAN [54] were used to construct trajectories on the imputed expression values. Monocle 2 uses a DDR-Tree (Discriminative DRTree) [83] for dimensionality reduction and tree construction. For TSCAN, we calculated the top principal components (PCs) with the prcomp function in the stats R package. We then run *k*-means clustering (using the kmeans function in the stats R package) on the top PCs (obtained by TSCAN using elbow method) to obtain predicted cluster labels for each cell. Then, we used the predicted cluster labels and top PCs as input to TSCAN. The number of clusters was chosen to be the smallest one that allowed two branches in the spanning tree to be consistent with the underlying true tree structure.

To identify the root cells or root state when inferring trajectories with TSCAN and Monocle 2 using CellBench data, the cluster with the most H2228 cells was selected. For *HCA_10x_tissue data*, each cell was assigned a differentiation level, as follows. HSC: level 1; MPP: level 2; LMPP and CMP: level 3; CLP, GMP, and MEP: level 4; B cell, CD4 T cell, CD8 T cell, NK cell, Monocyte, and Erythroid: level 5. Then, the cluster of cells with the smallest averaged differentiation level was set to be root state for the inferred trajectories.

#### Assessment

For the CellBench [18] data, we used the same performance metrics as in Tian et al.’s work [18]: (1) the Pearson correlation between the inferred trajectory and the rank order of the cells for which we know the true ordering of the cells, and (2) the proportion of cells on an inferred trajectory for which the inferred branch and the true branch were the same.

For the bone marrow cells from the *HCA_10x_tissue data*, we compared the estimated pseudotime to the level of differentiation for each pair of cells. For instance, for a pair of cells A and B, if cell A is a HSC (differentiation level 1), cell B is a MPP (differentiation level 2), and the inferred pseudotime for cell A and B is *t*_*A*_ and *t*_*B*_ where *t*_*A*_<*t*_*B*_, then we call it “correctly ordered.” The *percentage* of the correctly ordered cells averaged across all possible cell pairs served as the performance metric.

### Performance of imputation algorithms on time, memory, and scalability

We created four datasets for this analysis. Using the *10x_293t_jurkat* dataset, we created two smaller datasets by randomly sampling *N* = 1000 and *N* = 5 000 cells (*1k_cell*, *5k_cell*, respectively). Using the *HCA_10x_tissue* dataset, we created two larger datasets by randomly sampling *N* = 50,000 and *N* = 100,000 cells (*50k_cell*, *100k_cell*, respectively). By running the imputation methods on all datasets, we assessed the computational time (in minutes), memory usage (MaxRSS or maximum resident set size of all tasks in a job in gigabytes (GB) returned from the Slurm command sacct), and scalability with respect to cell number (Additional file 1: Figure S11, Additional file 6: Table S5). To describe scalability for each method, a linear model was fit using the lm function from the stats R package where the computation time was the response and the number of cells on the log10-scale was the predictor. The coefficient of the cell number represents the scalability of the method. For methods that failed to produce results, the running time was set to be the maximum time (72 h) plus 1 min, and the memory was set to be the maximum memory. The time and memory were linearly scaled to [0,1]. The average scaled time and memory across all datasets were used as the final score as shown in Fig. 6.

### Overall performance score

We have assessed the performance of 18 imputation methods in a total of 1100 evaluation settings: 30 in bulk correlation, 1032 in differential analysis (972 in ranking differentially expressed genes, 60 in null differential analysis), 6 in predicting cell types using PBMC data, 16 in clustering analysis, 12 in trajectory analysis, and 4 in efficiency assessment. All the assessment measures were mapped to [ 0,1] by subtracting the theoretical minimum and then dividing by the difference of the theoretical maximum and minimum. Empirical extrema were used when theoretical ones did not exist. Ten thousand permutations was applied to obtain the empirical minimum of ARI for each dataset. *H*_*acc*_ and *H*_*pur*_ were further subtracted by 1 for the convenience of applying “the higher the score, the better the performance” criterion. Efficiency measures (time, memory, scalability) were scaled with the extrema of all methods’ performance.

Evaluation of imputed values through the similarity to bulk samples, differential analysis, clustering, and pseudotime inference were considered as four main assessment aspects. In each aspect, the scores across datasets were first averaged, and then the mean from different analysis tools (MAST and Wilcoxon; *k*-means and Louvain clustering; Monocle 2 and TSCAN) were averaged. Next, for each aspect except for clustering, the mean of multiple assessment statistics (e.g., overlap and correlation in assessing pseudotime inference) was used as the evaluation score. For clustering, since *H*_*acc*_,*H*_*pur*_ and ARI are highly correlated (Fig. 4a, d), we first computed the average of *H*_*acc*_,*H*_*pur*_ and ARI, and then averaged it with *medianSil* and used the final average as the evaluation score. This is to avoid the three highly correlated metrics (*H*_*acc*_,*H*_*pur*_ and ARI) dominating the evaluation. Finally, the mean evaluation scores of all four assessment aspects was used to derive methods’ overall performance rank.

## Supplementary information

**Additional file 1** **Supplementary Figures.** Supplementary Figures S1-S11. (PDF 17.4 MB).

**Additional file 2** **Supplementary Table S1.** Comparison of our benchmark with other benchmark analyses involving imputation methods. (XLSX 11KB)

**Additional file 3** **Supplementary Table S2.** Table of the Spearman correlation coefficients (SCC) shown in Fig. 2d, h filled circles between the number of cells and the correlation scores. We used the Spearman correlation coefficients (SCC) of an imputation method (values in heatmap Fig. 2d) and the number of cells in the columns as inputs to calculate the Spearman correlation between the number of cells and the scores. (CSV 1 KB)

**Additional file 4** **Supplementary Table S3.** Summary of all datasets used in each evaluation of this benchmark. The table includes the names, protocols, source (GEO accession numbers or links to download) and cell details of each dataset. (XLSX 16 KB)

**Additional file 5** **Supplementary Table S4.** Summary of all scRNA-seq imputation methods used in each evaluation of this benchmark. The table includes the name of the method, input, output, pre-processing steps for each method that we applied, the programming language, assumptions about the method, the download date, software version number, and link to software package. (XLSX 15KB)

**Additional file 6** **Supplementary Table S5.** Values of all three efficient measures in time, memory and scalability using all four datasets. The table include the computation time and memory of four datasets with 10^3^,5×10^3^,5×10^4^,10^5^ cells for all imputation methods. Scalability is the coefficient of the cell number of each dataset in the linear model where the number of cells on the log10-scale is fitted against the computation time. (CSV 3 KB)

**Additional file 7** Review history.

## Data Availability

The data used in this analysis are all publicly available. All data are described in the “Methods” section and Additional file 4: Table S3 with all links or GEO accession numbers. The imputation methods are described in Additional file 5: Table S4. All code to reproduce the presented analyses are available at https://github.com/Winnie09/imputationBenchmark[84]. The version of source code used in this article was deposited in Zenodo with the access code DOI: 10.5281/zenodo.3967825 (10.5281/zenodo.3967825) [85]. The R package ggplot2 [86] for data visualization was used. All accession numbers are listed in Additional file 4: Table S3, but we list them here too: GSE81861 [17], GSE118767 [18], https://support.10xgenomics.com/single-cell-gene-expression/datasets[3], https://preview.data.humancellatlas.org/[11], GSE86337 [69], GSE129240 [72], and GSE74246 [73]. Other bulk RNA-seq samples are from ENCODE [71].
