# Supplementary material for: A systematic evaluation of single-cell RNA-sequencing imputation methods
Source: Genome Biol. 2020 Aug 27;21:218. doi: 10.1186/s13059-020-02132-x (PMC7450705; doi:10.1186/s13059-020-02132-x)
Supplement: Supplementary file 1 — Additional file 1 Supplementary Figures. Supplementary Figures S1-S11. (PDF 17.4 MB). [file 13059_2020_2132_MOESM1_ESM.pdf]

Additional file 1

Supplementary Figures

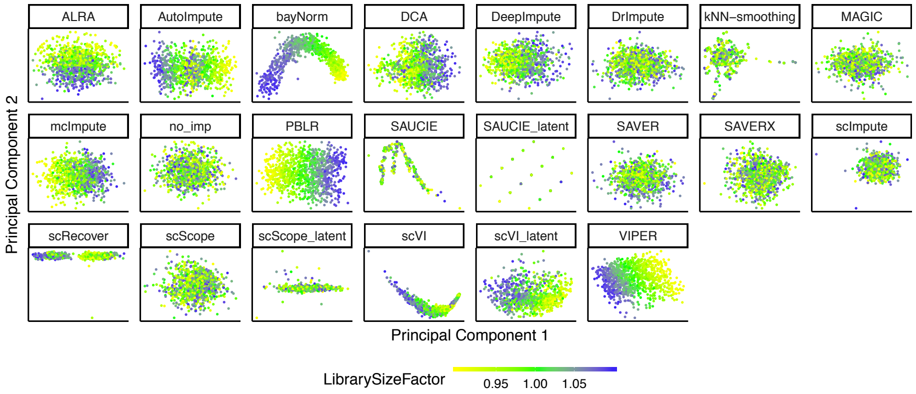

Figure S1 Dimension reduction results after applying Principal Component Analysis (PCA) from either no imputation (*no_imp*) or the 18 imputation methods using the *null simulations* data, except the difference between this figure and Figure 1A is this figure includes the latent spaces directly found by scScope (scScope_latent), scVI (scVI_latent) and SAUCIE (SAUCIE_latent) (not Principal Components – PCs). All other methods are showing observations along the first two PCs. The color represents the simulated library size (defined as the total sum of counts across all relevant features) for each cell.

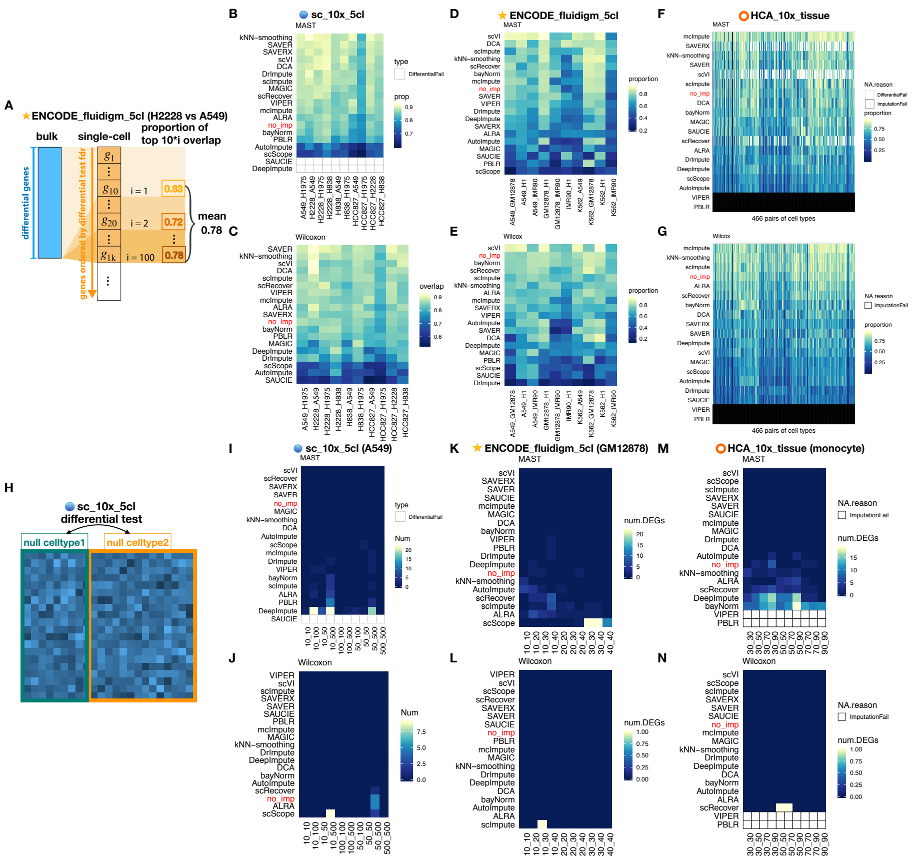

Figure S2 Impact of imputation methods on differential expression analysis. **(A)** Schematic of evaluating differentially expressed genes (DEGs) using the overlap between bulk RNA-seq and scRNA-seq – also shown in Figure 2A. Using the pairs of cell lines in the *sc_10x_5cl* dataset, *ENCODE_fluidigm_5cl* dataset, and pairs of cell types in the bone marrow tissue from the *HCA_10x_tissue* dataset, we show heatmaps of proportion of overlap between bulk and single-cell DEGs identified using **(B, D, F)** MAST [1] and **(C, E, G)** Wilcoxon-rank-sum test [2] (abbreviated as Wilcoxon) for differential expression analysis, respectively. **(H)** Schematic of a null differential expression analysis by randomly partitioning cells from the same cell type into two groups – also shown in Figure 2E. Using the A549 cells from the *sc_10x_5cl* dataset, the GM12878 cells from the *ENCODE_fluidigm_5cl* dataset, and bone marrow cells from the *HCA_10x_tissue* dataset, the number of false positive DEGs identified using **(I, K, M)** MAST and **(J, L, N)** Wilcoxon, respectively. The x-axis in Figures (I-N) describe the number of cells in each group (e.g. 10 sampled cells in group 1 and 10 sampled cells in group 2) when applying a method to identify differentially expressed genes. White areas with black outline indicate that the imputation methods did not return output after 72 hours and areas with grey outline indicate that either MAST or Wilcoxon failed to return results.

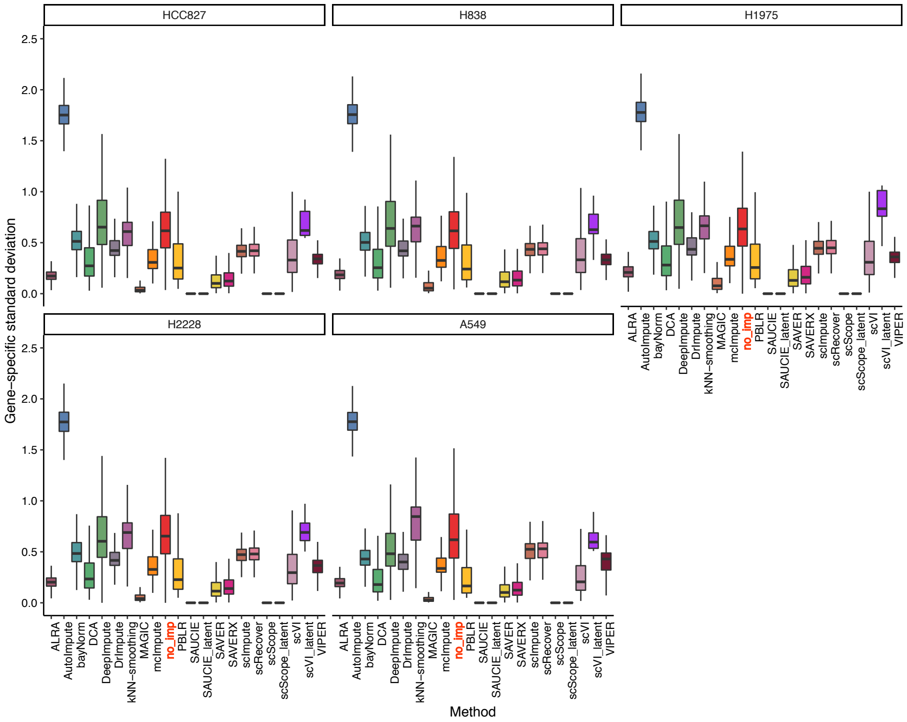

Figure S3 Estimates of gene-specific standard deviation of imputed values for five cell lines. For each cell line in the *sc_10x_5cl* dataset, we calculated the gene-specific standard deviation across cells with no imputation (*no_imp*) and with imputation.

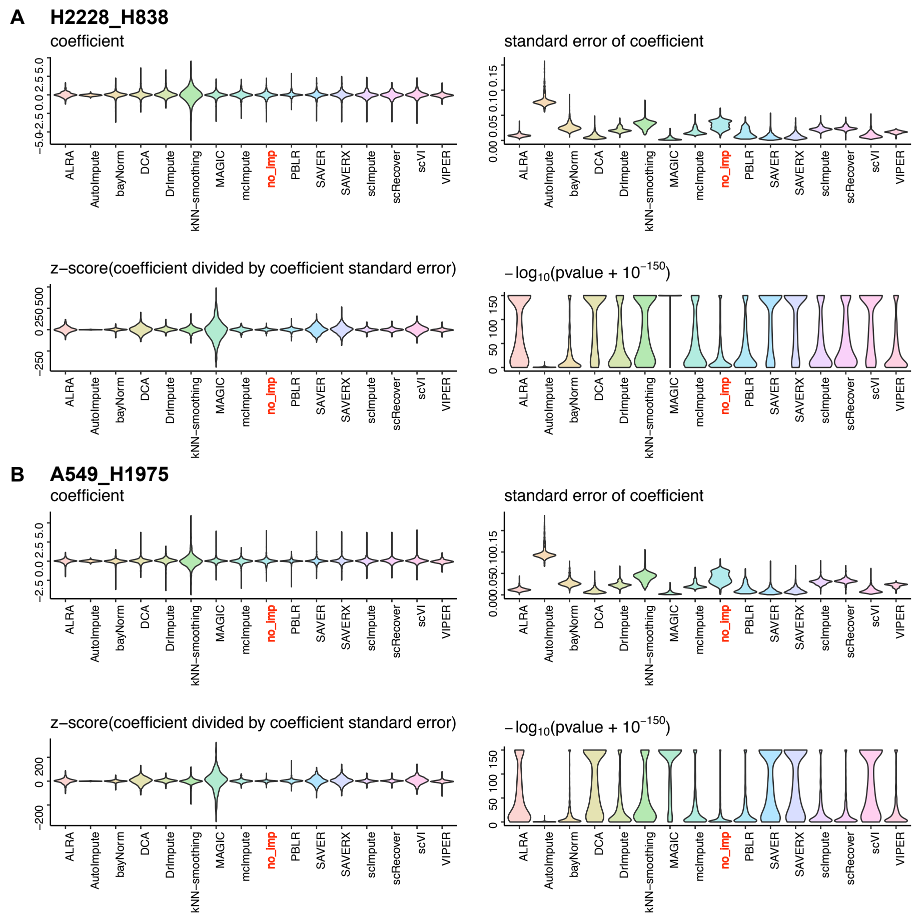

Figure S4 Summary distributions of output from MAST using the imputed values from pairs of cell lines. The pairs of cell lines from the *sc_10x_5cl* dataset compared below are **(A)** H2228 (N=758) vs H838 (N=876) – more balanced group sizes and **(B)** A549 (N=1256) vs H1975 (N=440) – less balanced group sizes. For each pair of cell lines, we applied MAST and extracted and plot the following information: the distribution of estimated log-fold changes ('coefficients') (top left), the distribution of standard errors for the coefficients (top right), the distribution of test statistics (z-score output from MAST, bottom left), and distribution of log-transformed p-values (bottom right).

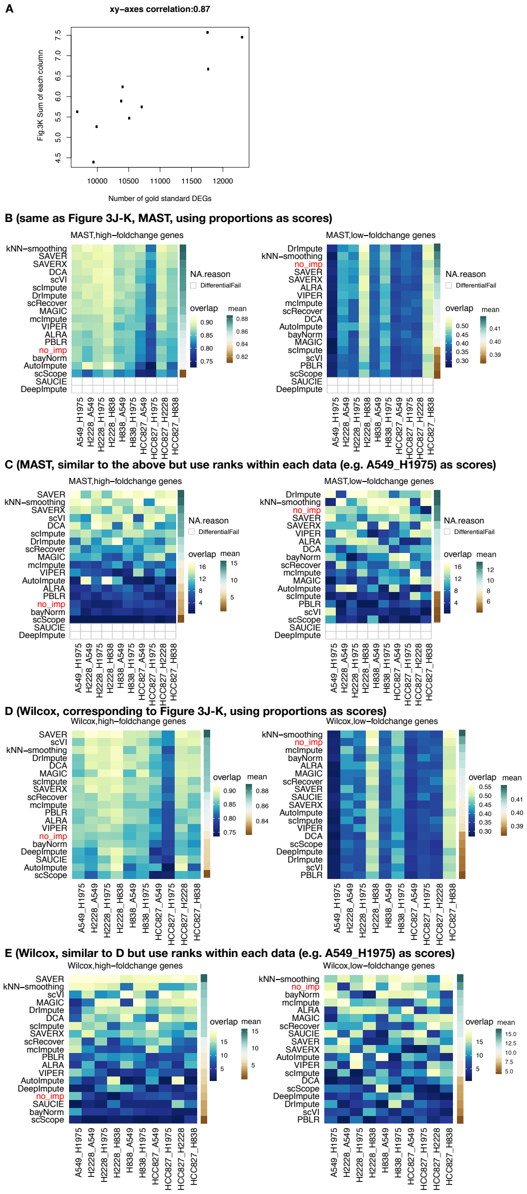

Figure S5 Results for Figure 3J and 3K that are adjusted for differences in the number of "gold standard" (bulk) differentially expressed genes. (A) Using results in Figure 3K, the number of "gold standard" differentially expressed genes (DEGs) is related to the sum for each column. For example in H2228_H838 cell types, there are 12312 gold standard DEGs, therefore, when we only look at the low log-fold change genes (Figure 3K), many low log-fold-change genes will overlap with the gold standard already, no matter what imputation method has been performed. **(B)** Same figure as Figure 3J-K, reproduced here for comparison with (C). This heatmap shows the percentage of the overlap between bulk and single-cell DEGs identified using MAST stratified by genes with high (top 10%) or low (bottom 10%) log-fold changes. The color bar on the last column shows the mean overlap across all comparison for each method. If MAST failed to identify DEGs from the imputed profiles of any method in any dataset, we denoted it as "DifferentialFail". **(C)** Similar to (B), but to adjust for unwanted this data set specific variability, we use "ranks", which should not be affected by data set variability. First, we rank all methods based on the overlap proportion within each set of data (i.e. a pair of cell types, for example A549_H1975). The ranks should not depend on the set of data, so there is no variability across sets of data (see in the heatmap rows). Instead of averaging the overlap proportions as what we showed on main manuscript Figure 3J-K ((B) here), now we use these "ranks" as scores and averaged across datasets. We can see that the ranking between (B) and (C) are quite consistent. It tells that the methods variability is not affected by the dataset variability. **(D) (E)** Similar to the idea of showing (B) and (C) here but instead of using MAST we use Wilcoxon rank-sum test as the test method to identify DEGs.

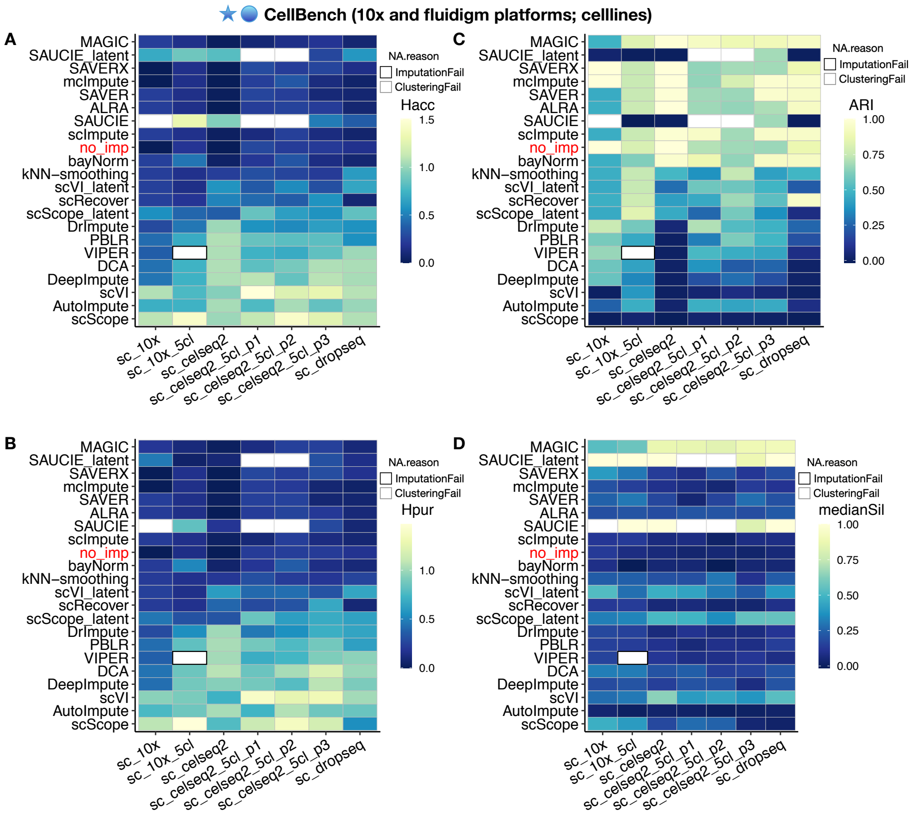

Figure S6 Impact of imputation methods on k-means clustering analysis using seven datasets from CellBench single cells. Heatmaps of the individual performance metrics **(A)** entropy of cluster accuracy (H_{acc}), **(B)** entropy of cluster purity (H_{pur}), **(C)** adjusted Rand index (ARI), and **(D)** the median of Silhouette and median Silhouette index of each imputation method for each of the seven datasets in CellBench [3]. The white boxes with black lines represent cases in which no output was returned from the imputation method after 72 hours. The white boxes with gray lines represent cases in which the clustering algorithm failed to cluster the cells using the principal components, for instance "more cluster centers than distinct data points" because many cells with imputed profiles are identical.

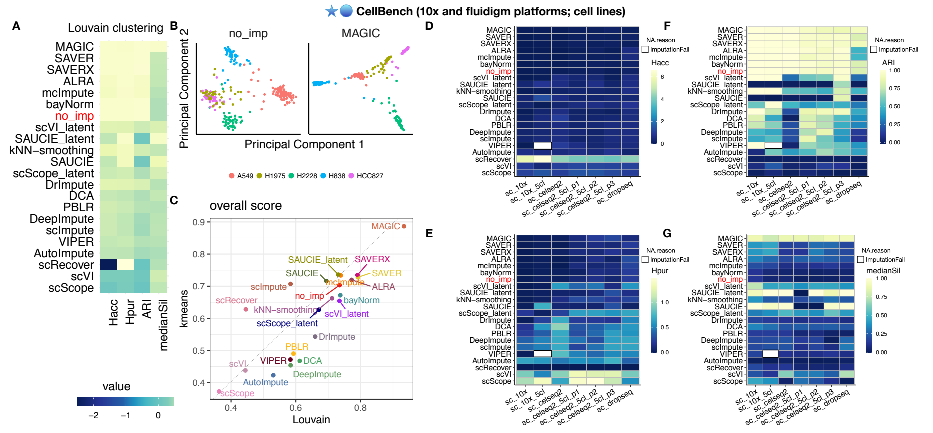

Figure S7 Impact of imputation methods on Louvain clustering analysis using seven datasets from CellBench [3]. **(A)** Heatmap of four performance metrics – entropy of cluster accuracy (H_{acc}), entropy of cluster purity (H_{pur}), adjusted Rand index (ARI), and median Silhouette index – averaged across seven datasets. To compare imputation methods across metrics, the metrics were re-scaled to be between 0 and 1 and the order of H_{acc} and H_{pur} were flipped to where a higher standardized score translates to better performance. Imputation methods (rows) are ranked by the average across all four metrics. **(B)** Dimension reduction results after applying PCA to the *sc_celseq2_5cl_p1* data with no imputation (left) and with imputation using MAGIC (right). The colors are the true group labels. **(C)** Overall score (or average of the four performance metrics) for Louvain clustering (x-axis) and k-means clustering (y-axis). **(D-G)** Heatmaps of the individual performance metrics (D) H_{acc}, (E) H_{pur}, (F) ARI and (G) the median of Silhouette of each imputation method for each CellBench dataset. The white boxes with black lines represent cases in which no output was returned from the imputation method after 72 hours.

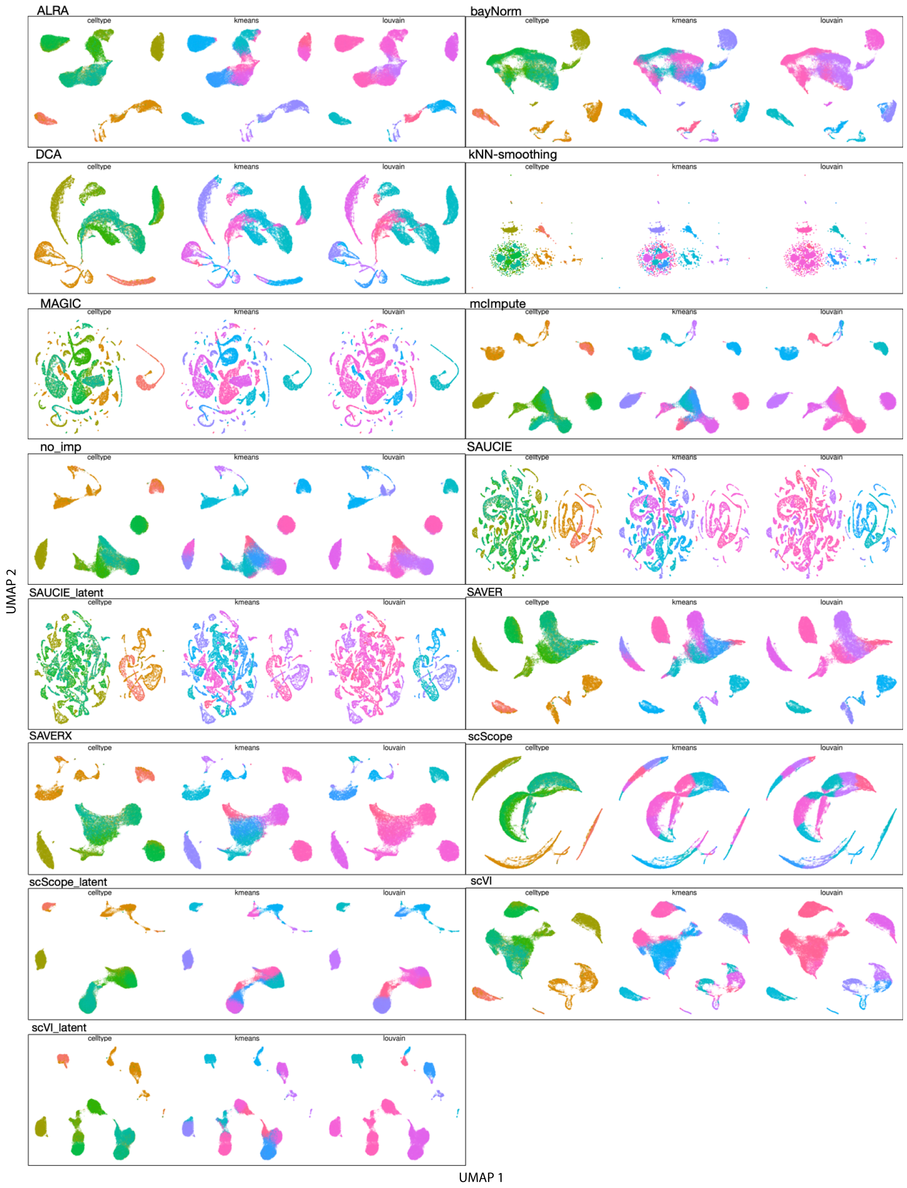

Figure S8 UMAP of the imputation methods output in clustering analysis using ten sorted peripheral blood mononuclear cell (PBMC) cell types from 10x Genomics. Dimension reduction results using UMAP components [4] derived from top principal components of imputed gene expression profiles (marked with the imputation method name) or from latent space representation (marked with '_latent'). For each method, there are three subplots where each dot is a cell, x-axis is UMAP coordinate 1 and y-axis is UMAP coordinate 2. The only difference of the three subplots is the way the dots are colored: colored by known cell types of the cells (left, titled "celltype"), colored by k-means clustering clusters (middle, titled "kmeans"), and colored by Louvain clustering clusters (right, titled "louvain"). This figure visualizes the unsupervised clustering results of each of the imputation methods.

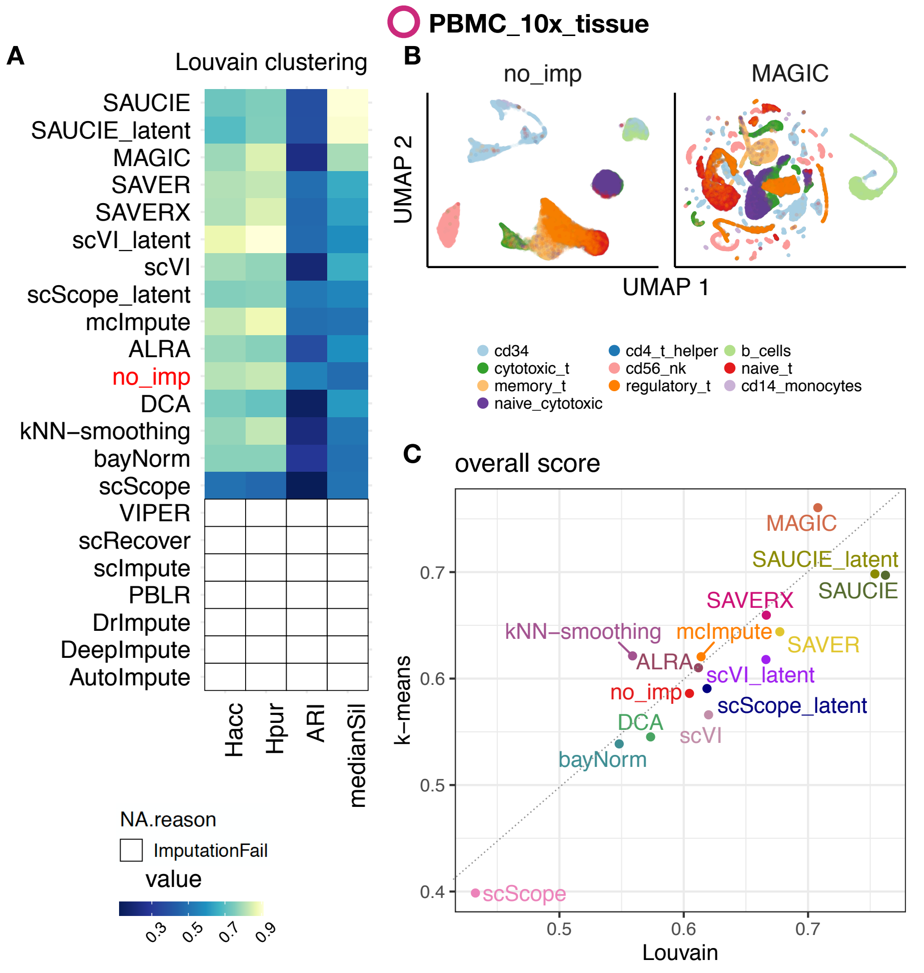

Figure S9 Impact of imputation methods on Louvain clustering analysis using ten sorted peripheral blood mononuclear cell (PBMC) cell types from 10x Genomics. **(A)** Heatmap of four performance metrics – entropy of cluster accuracy (H_{acc}), entropy of cluster purity (H_{pur}), adjusted Rand index (ARI), and median Silhouette index – on data from 10x Genomics [5]. To compare imputation methods across metrics, the metrics were re-scaled to be between 0 and 1 and the order of H_{acc} and H_{pur} were flipped to where a higher standardized score translates to better performance. Imputation methods (rows) are ranked by the average across all four metrics. **(B)** Dimension reduction results using UMAP components [4] with no imputation (left) and with imputation using MAGIC (right). The colors are the true group labels. **(C)** Overall score (or average of the four performance metrics) for Louvain clustering (x-axis) and k-means clustering (y-axis). White areas with black outline in (D) indicate that the imputation methods did not return output after 72 hours.

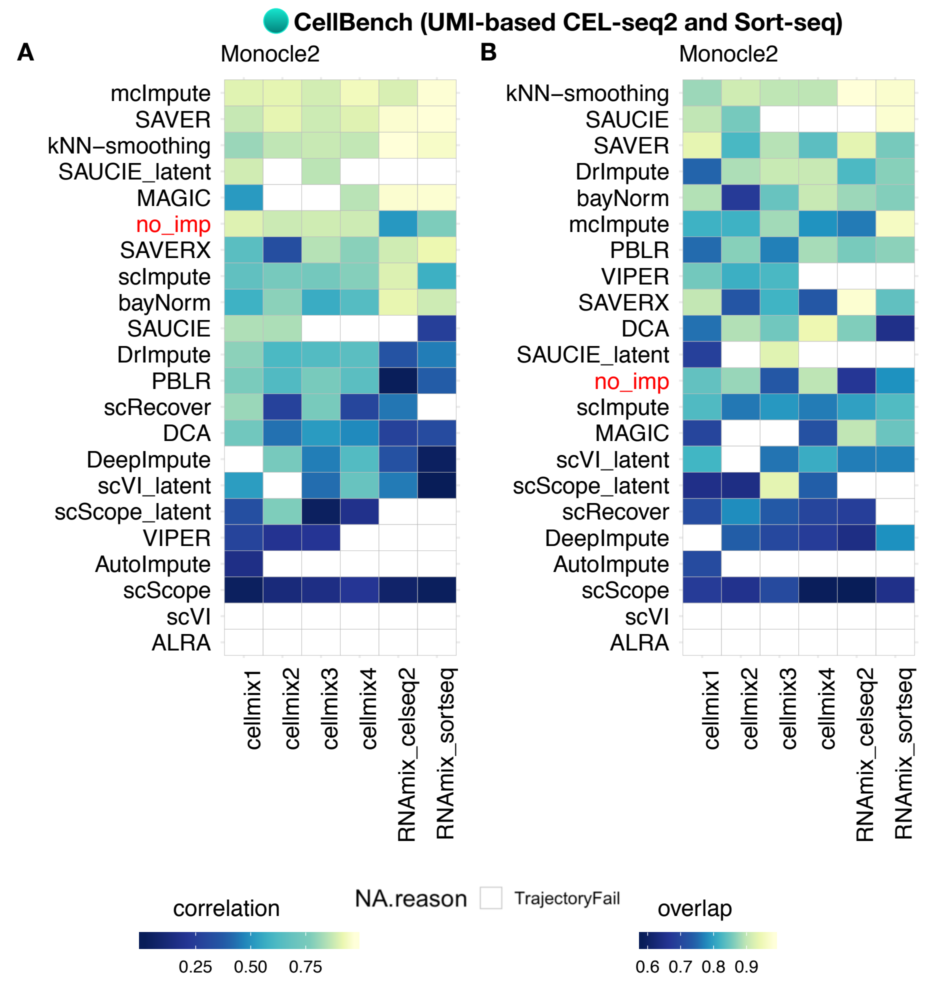

Figure S10 Impact of imputation methods on inferred trajectories for pseudotime analysis using Monocle 2 with the RNA mixture and cell mixture datasets from CellBench [3]. (A) Heatmap showing the Pearson correlation coefficients (PCC), denoted as *correlation*, between the inferred trajectory and the rank order of the cells where we know the true trajectory (or ordering) of the cells. **(B)** Heatmap of the proportion of cells on the inferred trajectories that correctly *overlap* with the cells on the branch where we know the true trajectory of the cells.

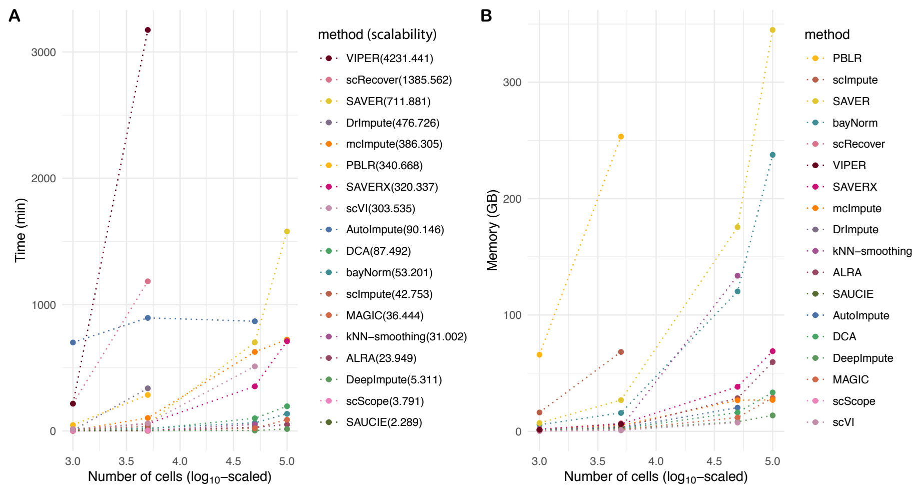

Figure S11 Values of time, scalability and memory of each imputation method on each dataset before scaling. **(A)** Computation time (in minutes) for each method to finish imputing four datasets of 10^3, 5×10^3, 5×10^4 and 10^5 cells, respectively. For some imputation methods, no results are shown because no imputed values were returned within 72 hours. Scalability (marked in the parentheses after each methods's name) is defined by fitting a linear model with the number of cells on the $\log_{10}$-scaled on the x-axis and the computation time on the y-axis and using the coefficient as the metric for scalability. **(B)** Memory usage (in maximum resident set size of all tasks in job (MaxRSS) in gigabyte (GB), returned from the Slurm command `sacct`) for each method to finish imputating the four datasets mentioned above.

Author details

References

1. Finak, G., McDavid, A., Yajima, M., Deng, J., Gersuk, V., Shalek, A.K., Slichter, C.K., Miller, H.W., McElrath, M.J., Prlic, M., *et al.*: Mast: a flexible statistical framework for assessing transcriptional changes and characterizing heterogeneity in single-cell rna sequencing data. Genome biology **16**(1), 278 (2015)
2. Bauer, D.F.: Constructing confidence sets using rank statistics. Journal of the American Statistical Association **67**(339), 687–690 (1972)
3. Tian, L., Dong, X., Freytag, S., Lê Cao, K.-A., Su, S., JalalAbadi, A., Amann-Zalcenstein, D., Weber, T.S., Seidi, A., Jabbari, J.S., Naik, S.H., Ritchie, M.E.: Benchmarking single cell RNA-sequencing analysis pipelines using mixture control experiments. Nat Methods **16**(6), 479–487 (2019). doi:10.1038/s41592-019-0425-8
4. Leland McInnes, J.M. John Healy: UMAP Uniform Manifold Approximation and Projection for Dimension Reduction. (2018). https://arxiv.org/abs/1802.03426
5. Zheng, G.X.Y., Terry, J.M., Belgrader, P., Ryvkin, P., Bent, Z.W., Wilson, R., Ziraldo, S.B., Wheeler, T.D., McDermott, G.P., Zhu, J., Gregory, M.T., Shuga, J., Montesclaros, L., Underwood, J.G., Masquelier, D.A., Nishimura, S.Y., Schnall-Levin, M., Wyatt, P.W., Hindson, C.M., Bharadwaj, R., Wong, A., Ness, K.D., Beppu, L.W., Deeg, H.J., McFarland, C., Loeb, K.R., Valente, W.J., Ericson, N.G., Stevens, E.A., Radich, J.P., Mikkelsen, T.S., Hindson, B.J., Bielas, J.H.: Massively parallel digital transcriptional profiling of single cells. Nat Commun **8**, 14049 (2017). doi:10.1038/ncomms14049
