# Supplementary material for: A systematic evaluation of single-cell RNA-sequencing imputation methods
Source: Genome Biol. 2020 Aug 27;21:218. doi: 10.1186/s13059-020-02132-x (PMC7450705; doi:10.1186/s13059-020-02132-x)
Supplement: Supplementary file 7 — Additional file 7 Review history. [file 13059_2020_2132_MOESM7_ESM.docx]

Review History

**First round of review**

**Reviewer 1**

**Are you able to assess all statistics in the manuscript, including the appropriateness of statistical tests used?**

Yes, and I have assessed the statistics in my report.

**Comments to author:**

Accessibility to single-cell RNA-sequencing technologies has necessitated the development of many computational methods for data analysis, including imputation methods that aim to recover missing expression information common to scRNA-seq data. Here, Hou et al evaluate 18 (and later 21) scRNA-seq imputation methods using a series of thorough benchmark analyses with real empirical scRNA-seq and bulk RNA-seq datasets.

It is evident that a lot of work and analysis has gone into this paper. However, many of the analyses would greatly benefit from further clarification as to the motivation for the analysis, why certain choices were made in terms of metrics/statistics, and a more in-depth interpretation of the findings. The paper unfortunately leaves readers with very few concrete and actionable takeaways, thus limiting its impact.

The paper is not suitable for publication in its current form. I hope these comments will be helpful to the authors moving forward.

---

Due to the large number of comments, I have organized them as corresponding to particular results in the Results Sections.

\## 2.1 Similarity between bulk RNA-seq and imputed scRNA-seq data

1. The authors provide a systematic benchmark comparison and evaluation of 18 scRNA-seq imputation methods noted in Figure 1B and lines 55-59. The paper would greatly benefit from a brief overview of each the tested method, highlighting their key similarities and differences. As newer imputation methods are rapidly being developed, it will be important to give readers general concepts and takeaways that may still be applicable to these newer methods.

2. The authors note that "Methods such as SAVER and SAVER-X (without pre-training) performed well with 10x Genomics UMI count data, but their performance gain was not as pronounced with read count data from the plate-based Fluidigm platform, as expected" (page 3, line 33). Why is this expected? Please elaborate. Along the same lines, what does the authors mean that "the performance gain was not as pronounced"? Could the authors provide a more quantitative characterization of this observation?

3. The authors note that "methods such as scVI, DCA...performed well in both settings" (page 3, line 34) meaning both 10x and Fluidigm platforms. However it seems like most methods, including scVI and DCA, performed better with the plate-based Fluidigm platform based on the heatmap in Fig. 2D. The difference in performance between platforms could be statistically evaluated to provide a more quantitative assessment of the performance difference of these imputation methods across platforms.

4. The authors assess each imputation method's ability to preserve the difference on the log scale between two cell-type profiles. If my understanding is correct, the authors are looking at each imputation method's ability to preserve the log fold changes when comparing two cell-types in single cell compared to bulk. The axes in Figure 2E-F should be fixed to note that these values are log fold changes.

5. The authors note that "the performance of some imputation methods was found to be affected by the number of cells in the dataset with a smaller number of observations" (page 3, line 44). Results supporting this claim are not shown and could be added to Figure 2. The columns of the heatmaps could also be ordered by number of cells rather than alphabetically to emphasize this trend.

6. Generally speaking, can the authors comment on why the Spearman cross correlation on the imputed data is worse than a simple pseudobulk (as seen in Fig 2A-B, and E-F)? Doesn't this suggest that readers shouldn't bother with imputation and instead perform our differential expression analysis on pseudobulks?

\## 2.2 Impact of scRNA-seq imputation on identifying differentially expressed genes

1. It is unclear why the authors used both MAST and Wilcoxon rank-sum tests for single-cell differential expression. Figures 3B-C suggests that these two methods give consistent results. How are the two tests different? Do they provide orthogonal metrics for evaluation that provide different information?

2. The authors note that they "found smoothing-based (e.g. kNN-smoothing) and data reconstruction methods (e.g. scVI) had the highest overlap with bulk DEGs" (page 4, line 56). But it is not clear to a general reader which methods they are referring to unless the reader already has a solid understanding of each of the 18 methods and which use smoothing and which use data reconstruction.

3. The axes labels of figure 3F-H are described as "the number of false positive DEGs" (line 43). However, the axes range from negative to positive and does not match the description. I am unable to figure out what these axes are. Most of the data points are also not legible due to being at (0,0).

4. The authors note that "when the LFC was small, only 2/14 methods had large overlap between bulk and single-cell DEGs compared to no imputation, suggesting that more imputation methods may have smoothed small differential signals away" (page 6, line 19). However, this observation is based on the average degree of overlap across a number of arbitrarily chosen datasets. Based on Figure 3K, all methods perform quite comparably within datasets. Likewise, all methods perform quite variably across datasets. Conclusions based on the average performance across datasets seem poorly substantiated given this high variability.

5. The authors use both MAST and Wilcoxon tests for Figure 3B-D, F-H but switch to only MAST for I-K, even though MAST seems to fail for some methods. Is there a reason for this choice? Is there a reason for the failure? If MAST failed to identify any DEGs, should the overlap be 0? Also, does this mean the data points in Figure 3F-H for VIPER, scRecover, SAUCIE, DeepImpute that appear to be at 0 on the MAST axis are actually failures?

\## 2.3 Impact of scRNA-seq imputation on unsupervised clustering

1. Again, it is unclear why the authors used both k-means and Louvain clustering. Figures 4C and F suggests that these two methods give consistent results. How are the two methods different? Do they provide orthogonal metrics for evaluation that provide different information?

2. The authors use two metrics to assess clustering performance. However, to create an overall score, these four metrics are averaged, which does not seem appropriate given the high correlation between Hacc, Hpur, and ARI (e.g. the metrics are not independent and rather seem redundant). MedianSii also shows opposing trends to the other 3 metrics according to Figure 4.

3. The figures refers to entropy of cluster accuracy as Hacc and entropy of cluster purity as Hpur while the main text refers to entropy of cluster accuracy as ECA and entropy of cluster purity as ECP. Please be consistent in your use of acronyms.

4. Based on these performance metrics, MAGIC has consistently the highest overall score. However, in Figure 4E, the UMAP plot from MAGIC shows a large number of tiny spurious clusters and odd 'snakes' that seem to visually suggest that MAGIC is actually performing poorly. In particular, a biologist looking at that MAGIC UMAP plot might be inclined to incorrectly interpret each of those tiny clusters as a distinct cell-type. How have the authors evaluated whether their chosen set of statistics are properly capturing the accuracy of the unsupervised clustering? It may be useful to readers for UMAP plots of outputs from the other imputation methods to also be provided in the supplements.

\## 2.4 Impact of scRNA-seq imputation on inferring pseudotemporal trajectories

1. The authors expand the analysis here to 21 scRNA-seq imputation methods. What are these 3 new scRNA-seq imputation methods now included (they are not explicitly named in the text)? It is also unclear why this choice was made; why were previous analyses not performed with all 21 scRNA-seq imputation methods? Were the 3 new scRNA-seq imputation methods specific for pseudotemporal trajectory inference?

2. In Figure 6, the heatmap includes time, memory, and scalability. It is unclear what scale time and memory are on.

\## Conclusion/Overall

1. The authors conclude that the performance of these imputation methods depends on many factors including the experimental protocol, the sparsity of the data, the number of cells in the dataset, and the magnitude of the effect sizes. But it is not clear how these factors, either individually or in combination, impact each imputation method. For example, if I am using the Fluidigm system with a few cells but am looking for large magnitude effect sizes, which method should I use? Or if I am using the 10X system with millions of cells but high sparsity, which method should I use? Or is no imputation the safest choice?

2. How do the authors' findings compare to Tian et al (mentioned in the paper) and other benchmarking analyses such as Andrews and Hemberg (2019) or Zhang and Zhang (2018)? If there are inconsistencies between these different benchmarking papers, the authors need to discuss why these inconsistencies arise and why their benchmarking provides a more valid performance assessment for these imputation methods. If they are highly consistent, the authors need to justify why their benchmarking represents a significant advance over these previously published studies.

3. While this topic remains of outsanding interest to a broad audience of biologists, the way the paper is currently written would be difficult for such a broad audience of biologists to understand. Many of the analyses would greatly benefit from further clarification as to the motivation for the analysis, why certain choices were made in terms of metrics/statistics, and a more in-depth interpretation of the findings.

**Reviewer 2**

**Are you able to assess all statistics in the manuscript, including the appropriateness of statistical tests used?**

Yes, and I have assessed the statistics in my report.

**Comments to author:**

accept with minor revisions (no re-review needed IMHO) because the field needs to see this paper -- a null simulation shows off how many methods are really just geared to make garbage data "look compelling". I was a little disappointed that the authors did not touch on RNA velocity, since both trajectory and transcriptome inference at present largely focus on the summed abundance of spliced and unspliced RNA -- this is particularly dumb since the correlation of a given cell at a given time point with its neighbors will be dominated by the spliced abundance, whilst the unspliced abundance (and more importantly, the ratio of the two) is almost certainly a better indicator of the cell's fate. However, integrating data from spliced and unspliced separately decouples this relationship, and as far as I know, nobody has really tried to separate the dual projections and reconcile them. So the idea of swapping out correlation-based trajectories for velocity-based trajectories ends up driving full speed into the brick wall of an open research problem.

As a bonus, without either spike-in cells (per the recent paper from Christoph Bock and colleagues) or spike-in RNA molecules representing both spliced and unspliced transcripts (per the recent SmartSeq3 paper), I'm not sure any of these things can be considered quantitative. (Trajectories in general can be thought of as a depiction of where the cells were perhaps 6 hours ago, which is a bit perverse considering how fast things like Wnt/Hedgehog planar cueing, JAK-STAT signaling, steroid receptors, or metabolic rewiring happen!)

But at the end of the day, this paper shows that most imputation methods are useless for deep inference *even when they successfully complete matrices equivalent to bulk* and neither improve lineage inference nor clustering, perhaps the two most common tasks in single-cell RNA sequencing analyses. I suspect the reason is very deep-seated and requires more thought than many groups seem willing to give it; happily, neither Dr. Hicks nor Dr. Ji's lab has ever been shy about tackling brutally difficult, unpopular problems (cf. BIRD).

I think this paper is of great interest to all readers who care about science instead of science fiction; both Ben Johnson and Pamela Himadewi in my lab agree, as they both contributed substantially to the content of this review.

Timothy J. Triche, Jr., PhD

**Authors Response**

**Point-by-point responses to the reviewers’ comments:**

Reviewer reports :

Reviewer #1 :

Accessibility to single-cell RNA-sequencing technologies has necessitated the development of many computational methods for data analysis, including imputation methods that aim to recover missing expression information common to scRNA-seq data. Here, Hou et al evaluate 18 (and later 21) scRNA-seq imputation methods using a series of thorough benchmark analyses with real empirical scRNA-seq and bulk RNA-seq datasets.

It is evident that a lot of work and analysis has gone into this paper. However, many of the analyses would greatly benefit from further clarification as to the motivation for the analysis, why certain choices were made in terms of metrics/statistics, and a more in-depth interpretation of the findings. The paper unfortunately leaves readers with very few concrete and actionable takeaways, thus limiting its impact.

The paper is not suitable for publication in its current form. I hope these comments will be helpful to the authors moving forward .

*We thank this reviewer for your invaluable feedback. We have carefully considered your suggestions and advice and addressed them in the revised manuscript as we will detail below.*

---

Due to the large number of comments, I have organized them as corresponding to particular results in the Results Sections.

\## 2.1 Similarity between bulk RNA-seq and imputed scRNA-seq data

1. The authors provide a systematic benchmark comparison and evaluation of 18 scRNA-seq imputation methods noted in Figure 1B and lines 55-59. The paper would greatly benefit from a brief overview of each the tested method, highlighting their key similarities and differences. As newer imputation methods are rapidly being developed, it will be important to give readers general concepts and takeaways that may still be applicable to these newer methods.

*We thank the reviewer’s comments. As suggested by the reviewer, we have now added a new subsection (4.1 Overview of scRNA-seq Imputation Methods) under the Methods section and Additional file 5 -- Supplementary Table S4 where we provide an overview of the tested methods and highlighting their key similarities and differences. This will give readers general concepts about these methods. With regard to takeaways, we did not find any single category of methods whose performance dominates the other categories. However, there were several important takeaways discussed in our Discussion section for new method developers. For example, we found that compared to no imputation, existing imputation methods generally are more helpful for analyzing individual genes rather than cell-to-cell relationships (i.e. cell clustering, trajectory, etc.). Thus, for new imputation method development, it is important to investigate ways to further improve the analysis of cell-to-cell relationship.*

2.	The authors note that "Methods such as SAVER and SAVER-X (without pre-training) performed well with 10x Genomics UMI count data, but their performance gain was not as pronounced with read count data from the plate-based Fluidigm platform, as expected" (page 3, line 33). Why is this expected? Please elaborate. Along the same lines, what does the authors mean that "the performance gain was not as pronounced"? Could the authors provide a more quantitative characterization of this observation?

*We have now clarified and expanded this sentence to provide a quantitative characterization and explanation for why this is expected in the following (Section 2.1):*

*“Methods such as SAVER and SAVER-X (without pre-training) performed well with 10x Genomics UMI count data (ranked 2 and 3 out of 18), but their performance gain was not as pronounced with read count data from the plate-based Fluidigm platform (ranked 12 and 13 out of 18) (Figure 2D). We note that this difference in performance is expected as SAVER and SAVER-X were designed for only data that follow a negative binomial distribution [20, 21]. UMI count data have been shown to follow a negative binomial distribution as opposed to read count data which have been shown to follow a zero-inflated negative binomial distribution [42, 43, 44, 45].”*

3.	The authors note that "methods such as scVI, DCA...performed well in both settings" (page 3, line 34) meaning both 10x and Fluidigm platforms. However it seems like most methods, including scVI and DCA, performed better with the plate-based Fluidigm platform based on the heatmap in Fig. 2D. The difference in performance between platforms could be statistically evaluated to provide a more quantitative assessment of the performance difference of these imputation methods across platforms.

*Thank you for pointing this out. We are sorry for not making this more clear, but what we intended to say is that “methods such as scVI, DCA performed better compared to other methods within each platform”. In other words, we were trying to compare different methods within each platform, and we did not intend to say that performance of these methods is similar when comparing across platforms. We have revised the original description in the manuscript as:*

*“Within each platform, we found that the methods scVI, DCA and MAGIC performed better compared to the other methods, and this was consistent in both the Fluidigm and 10x platforms (Figure 2D).”*

*However, you raise a good point on how different methods compare across platforms, which we did not discuss in the original submission. In the revised manuscript, we have added discussion about this difference. For a given row in Figure 2D, we use the Spearman’s correlation coefficient (SCC) scores (values in the heatmap) from an imputation method as input to a two-sample t-test to compare the performance between the Fluidigm and 10x datasets. The p -values are adjusted for multiple testing (Benjamini-Hochberg FDR method). We marked methods with significant platform differences, defined as FDR < 0.05 from two-sample t-tests and the relative performance change >25%, using asterisks on the right hand side of Fig. 2D and 2H. These provide a quantitative assessment of the performance difference of these imputation methods across platforms. In the revised Fig. 2D, two methods (PBLR, AutoImpute) showed significant cross-platform performance differences. In the revised Fig. 2H, nine methods (MAGIC, SAVERX, SAVER, kNN-smoothing, ALRA, scRecover, SAUCIE, scScope, AutoImpute) showed significant cross-platform performance differences.*

*In response to the changes made in Figures 2D and 2H, we have added the following text in the manuscript in Section 2.1, respectively:*

*“When comparing across platforms, PBLR and AutoImpute showed significant cross-platform performance differences (two-sample t-tests at a false discovery rate (FDR) < 0.05 and relative performance change |SCC 10x-SCC fluidigm | / max(SCC 10x ,SCC fluidigm ) > 25%). For both methods, the correlation between the imputed single-cell profile and bulk profile was higher in the Fluidigm platform than in the 10x platform (Figure 2D).”*

*“When comparing across platforms, 9 out of 18 imputation methods showed significant cross-platform performance differences (two-sample t-tests FDR<0.05, relative performance change>25%) (Figure 2H). Among these methods, AutoImpute and SAUCIE showed higher correlation between the imputed expression difference and the bulk expression difference in the Fluidigm platform, while the other methods (MAGIC, SAVERX, SAVER, kNN-smoothing, ALRA, scRecover, scScope) showed higher correlation in the 10x platform.”*

4.	The authors assess each imputation method's ability to preserve the difference on the log scale between two cell-type profiles. If my understanding is correct, the authors are looking at each imputation method's ability to preserve the log fold changes when comparing two cell-types in single cell compared to bulk. The axes in Figure 2E-F should be fixed to note that these values are log fold changes.

*We have now modified the axes in Figures 2E and 2F to note that these values are log fold changes. Specifically, we labeled the x and y axis in Figure 2E as “pseudobulk LFC” and “bulk (FPKM) LFC”, as well as those in Figure 2F as “scVI imputed cells LFC” and “bulk (FPKM) LFC”.*

5.	The authors note that "the performance of some imputation methods was found to be affected by the number of cells in the dataset with a smaller number of observations" (page 3, line 44). Results supporting this claim are not shown and could be added to Figure 2. The columns of the heatmaps could also be ordered by number of cells rather than alphabetically to emphasize this trend.

*We have now modified the order of the columns in the Figures 2D and 2H heatmaps so that the columns are ordered by decreasing numbers of cells. For Figure 2H, the number of cells here is defined as the minimum number of cells in the cell type pair (Figure 2H).*

*To quantify this trend, within each platform (10x or Fluidigm) and for each method we computed the Spearman correlation coefficient (SCC) between the method’s imputation performance (values in heatmap Figure 2D) and the cell number across datasets. Methods with correlation > 0.6 are marked using solid circles in Fig. 2D, where the green and brown circles correspond to 10x and Fluidigm platforms, respectively. We observe that a number of methods show high correlation between the method’s imputation performance and the cell number.*

*In Figure 2H, since there are two sets of cells (one set for each cell type), we considered the minimum number of cells in each pair of cell types and calculated the SCC of it with the scores in each row (method) for 10x and Fluidigm, separately. Analogous to Figure 2D, we marked methods with correlation>0.6 using filled circles in Figure 2H. The green and brown circles correspond to 10x and Fluidigm platforms. Again, we observe that there are some methods with high correlation between the method’s imputation performance and the cell number.*

*The reported correlation values are now included Additional file 3: Table S2. Finally, we discussed this in the main text in Section 2.1:*

*“Finally, the performance of some imputation methods were found to be affected by the number of cells in the dataset. For data generated using the same platform, we observed that a smaller cell number is associated with a smaller imputed value-bulk correlation for these methods (Figure 2D,H: green and brown dots on the right of the heatmaps; and Additional file 3: Table S2). For example, for Fluidigm datasets in Figure 2D, IMR90 with 23 cells showed smaller correlation compared to the other plate-based datasets with more cells. For methods such as scVI, DCA, MAGIC, scImpute, SAUCIE, PBLR and DeepImpute, the Spearman correlation between the cell number and performance (imputation-bulk SCC) across the Fluidigm datasets was higher than 0.6 (Figure 2D).”*

6. Generally speaking, can the authors comment on why the Spearman cross correlation on the imputed data is worse than a simple pseudobulk (as seen in Fig 2A-B, and E-F)? Doesn't this suggest that readers shouldn't bother with imputation and instead perform our differential expression analysis on pseudobulks?

*We thank the reviewer for this question. Bulk and pseudobulk both are averaging the expression profiles of many cells, and they both try to measure the cell population’s average behavior. In contrast, the imputed single-cell profiles try to describe the gene expression in each individual cell and hence are associated with higher variability due to cell-to-cell heterogeneity. Therefore, we expect the Spearman correlation coefficient (SCC) between a bulk and a pseudobulk to be higher than the SCC between a bulk and an imputed single-cell profile because the pseudobulk and bulk both average out the cell-to-cell heterogeneity. As demonstrated in previous literature, as one combines an increasing number of cells to create a pseudobulk sample, the pseudobulk sample will become increasingly more correlated with bulk sample (for example, [69-70]*). Thus, the pseudobulk used here is more like a reference for upper bound (or positive control) rather than lower bound (or negative control) of the imputation performance.*

*It is important to keep in mind that single-cell profiles can depict the variability in a cell population, but pseudobulk cannot characterize cell-to-cell variability. A rigorous differential expression analysis should detect differences between two cell types that cannot be explained by the background variation (including cell-to-cell variation). Thus pseudobulk cannot replace single-cell data in a rigorous differential expression analysis.*

*We have now discussed this in Methods Section 4.5.1:*

*“In the analyses above, pseudobulk was used as a reference for the approximate upper bound of the single-cell imputation performance since both bulk and pseudobulk profiles try to measure a cell population’s average behavior, and the correlation between a pseudobulk and the corresponding bulk profile is expected to increase as one pools an increasing number of cells to create the pseudobulk sample [69, 70]. Note that unlike single-cell profiles, pseudobulk cannot characterize cell-to-cell variability. Thus, pseudobulk cannot replace single-cell data in studies that require analyzing cell-to-cell variability, such as a rigorous differential expression analysis that aims to detect differences between two cell types that cannot be explained by the background cell-to-cell variation.”*

**[69]	Pliner, H.A., Packer, J.S., McFaline-Figueroa, J.L., Cusanovich, D.A., Daza, R.M., Aghamirzaie, D., Srivatsan, S., Qiu, X., Jackson, D., Minkina, A. and Adey, A.C., 2018. Cicero predicts cis-regulatory DNA interactions from single-cell chromatin accessibility data. Molecular cell, 71 (5), pp.858-871.*

**[70]	Zhou, W., Ji, Z., Fang, W. and Ji, H., 2019. Global prediction of chromatin accessibility using small-cell-number and single-cell RNA-seq. Nucleic acids research , 47(19), pp.e121-e121.*

\## 2.2 Impact of scRNA-seq imputation on identifying differentially expressed genes

1.	It is unclear why the authors used both MAST and Wilcoxon rank-sum tests for single-cell differential expression. Figures 3B-C suggests that these two methods give consistent results. How are the two tests different? Do they provide orthogonal metrics for evaluation that provide different information?

*We have now added the following text to the first paragraph in Section 2.2 to explain why we used both MAST and Wilcoxon tests and some important differences between them:*

*“We considered two methods for differential expression (DE) analysis (namely MAST [46] and Wilcoxon rank-sum test [47] abbreviated as Wilcoxon), because we wanted to assess whether the performance difference among the imputation methods in this section was consistent regardless of the choice of DE method or whether it was due to the choice of the DE method. While MAST uses a parametric model developed specifically for single-cell data, the Wilcoxon test is a non-parametric test commonly used for both bulk and single-cell data.”*

2.	The authors note that they "found smoothing-based (e.g. kNN-smoothing) and data reconstruction methods (e.g. scVI) had the highest overlap with bulk DEGs" (page 4, line 56). But it is not clear to a general reader which methods they are referring to unless the reader already has a solid understanding of each of the 18 methods and which use smoothing and which use data reconstruction.

*We agree with this comment. Our original text could be misleading since we did not find any single category of methods that dominates the other categories. Therefore, we have now modified the text to list the specific methods that performed best, while also noting which category each method belongs to:*

*“To summarize this DE enrichment analysis across the three datasets (sc_10x_5cl, ENCODE_fluidigm_5cl, HCA_10x_tissue) and two DE methods (MAST and Wilcoxon), broadly we found kNN-smoothing (a smoothing-based method) and scVI (a deep-learning method) had the highest overlap with bulk DEGs (Figure 3B-D), and they both most consistently outperformed no imputation (in 5 out of 6 test settings in Additional file 1: Figure S2B-G). The model-based methods SAVER and SAVER-X were among the top performers with UMI-based datasets, and they outperformed no imputation in 3 out of the 4 UMI-based test settings (Additional file 1: Figure S2B,C,F,G).”*

3.	The axes labels of figure 3F-H are described as "the number of false positive DEGs" (line 43). However, the axes range from negative to positive and does not match the description. I am unable to figure out what these axes are. Most of the data points are also not legible due to being at (0,0).

*We have now corrected the x and y axis range to begin at 0 in Figure 3F-H (to remove the negative range which does not contain any data points) and we have used a different approach to label the points on the plots. These axis labels represent the number of false positive DEGs averaged across all settings identified by MAST (x-axis) and Wilcoxon (y-axis). We have modified the Figures 3F-H as follows:*

*Since these plots show the average number of false positives introduced by the imputation methods, points near zero are good and far away from zero are worse. Therefore, the idea is that for the big clouds of points near (0,0), these are good and we do not necessarily differentiate the marginal difference among them. Our aim is to emphasize the points further away from (0,0). We incorporated this into the main text in Section 2.2:*

*“Broadly, we found the majority of methods perform well in this analysis (specifically most methods are close to the (0,0) coordinate in Figure 3F-H), but for the methods that identified false positive DEGs, we found no consistency in which types of imputation methods identified false positive DEGs as there were examples in all types of imputation methods (model-based, smoothing-based and data reconstruction based) that reported false positive DEGs (Additional file 1: Figure S2I-N).”*

*This explanation has also been updated in Figure 3 caption.*

*“(F-H) Number of false positive DEGs averaging across all settings identified by MAST (x-axis) or Wilcoxon rank-sum test (y-axis) in null differential analyses.”*

4. The authors note that "when the LFC was small, only 2/14 methods had large overlap between bulk and single-cell DEGs compared to no imputation, suggesting that more imputation methods may have smoothed small differential signals away" (page 6, line 19). However, this observation is based on the average degree of overlap across a number of arbitrarily chosen datasets. Based on Figure 3K, all methods perform quite comparably within datasets. Likewise, all methods perform quite variably across datasets. Conclusions based on the average performance across datasets seem poorly substantiated given this high variability*.*

*We appreciate the reviewer’s comment about performance variability within and across datasets in Figure 3K.*

*We are aware that there is high variability across datasets and this is because different datasets have different signal to noise ratio. For example, the number of gold standard bulk differential genes detected using the same analysis procedure varied substantially across datasets. Some datasets tend to have more differential genes and stronger differential signals, therefore all methods will tend to show better DE analysis performance in those datasets. In order to show this, we have done an extra analysis as follows.*

*Regarding Figure 3K, which has high cross-dataset variability, the column sums (i.e., global performance of all imputation methods) is highly correlated with the number of gold standard differentially expressed genes (DEGs), as shown in the following figure to the right. For example in H2228_H838 cell types, there are 12312 gold standard DEGs, therefore, when we only look at the low log-fold change genes (Figure 3K), many low log-fold-change genes will overlap with the gold standard already, no matter what imputation method has been performed.*

*Despite this high variability across datasets, this source of variability is less of a concern in our benchmark because our primary goal is to compare different methods conditional on applying them to the same dataset. In other words, there are two sources of variability in Figures such as Fig. 3K. One is method variability due to performance difference. The other is dataset variability as we mention above. They are not in conflict with each other. In real applications, whether a biological system has strong or weak differential expression (i.e., dataset variability) often is determined by biology and cannot be controlled by a researcher. Thus, in this benchmark study, we are primarily interested in method variability, and we carefully account for dataset variability by comparing the methods on the same sets of data. Note that within each dataset, methods can be meaningfully compared. Conceptually, as long as a method A consistently performs better than another method B in most datasets, one can say that method A has overall better performance than method B regardless of how big the dataset variability is in Fig. 3K. In Figure 3J and 3K, a method’s overall performance is characterized using its average performance across all datasets. Since all methods are applied to the same sets of data, their overall performance can be meaningfully compared to determine their relative rank. We acknowledge that due to large dataset variability, Figure 3J and 3K may not show methods’ relative performance within each dataset clearly. Therefore, in order to more clearly show methods’ relative performance within each dataset, we have added a new analysis that shows methods’ ranks within each dataset (Additional file 1: Figure S5C,E), and we incorporated this analysis to the new Additional file 1: Figure S5. The overall conclusions remained largely the same.*

*Below we describe the new Additional file 1: Figure S5 in detail.*

*Additional file 1: Figure S5A is the plot above that shows the relationship between the number of gold standard DEGs in a dataset and the global performance of all imputation methods in the same dataset (i.e. the sum along the columns in Figure 3K).*

*Additional file 1: Figure S5B is the same as Figures 3J and 3K in the main manuscript (using MAST). We placed it here to make it easy to compare with Figure S5C.*

*Additional file 1: Figure S5C is similar to Additional file 1: Figure S5B, adjusted for unwanted data set specific variability by showing methods’ “ranks” (using MAST). Specifically, we first rank all methods based on the overlap proportion within each set of data (i.e. a pair of cell types, for example A549_H1975), and then show the ranks in the heatmap. Since ranks have the same range and distribution in all datasets, examining methods’ relative performance by ranks is less influenced by dataset variability. Indeed, the method variability is more pronounced than the dataset variability in Figure S5C. In this figure, we used these “ranks” as scores and averaged them across datasets to give each method an overall performance score, and methods were then ranked based on this overall score (Additional file 1: Figure S5C). One can see that the method ranking between Additional file 1: Figures S5B and S5C are largely consistent. Importantly, in both figures, one can see that when LFC is small, only very few methods outperformed no imputation. By contrast, a large number of methods outperformed no imputation when LFC is large.*

*Additional file 1: Figures S5D and S5E -- Analogously, we performed the same analysis, but using the Wilcoxon rank-sum test and observed the same pattern.*

5. The authors use both MAST and Wilcoxon tests for Figure 3B-D, F-H but switch to only MAST for I-K, even though MAST seems to fail for some methods. Is there a reason for this choice? Is there a reason for the failure? If MAST failed to identify any DEGs, should the overlap be 0? Also, does this mean the data points in Figure 3F-H for VIPER, scRecover, SAUCIE, DeepImpute that appear to be at 0 on the MAST axis are actually failures?

*We have provided the results from both MAST and Wilcoxon for all analyses summarized in Figure 3 (B-D, F-H, J-K). Previously, the results for both MAST and Wilcoxon were included in the prior submission of this manuscript in Additional file S1: Figure S2 for Figure 3 B-D, F-H. Now we have added the results for Wilcoxon specifically for Figure 3J-K. These new results can be now found Additional file 1: Figure S5.*

*Figure 3I is not about differential test (Wilcoxon/MAST) results, but a heatmap of area under the receiver operating characteristic (ROC) curve (AUC) values when using the expression level of a marker gene (e.g. CD19) to predict a cell type (e.g. B cell or not) using UMI-based sorted PBMC cell types. Through this analysis we found that imputation methods generally help a lot in recovering the marker genes’ signals. This is not a traditional differential analysis but it is similar because we aim to use imputed values of marker genes to discriminate cell types, therefore we included this analysis in Figure 3. Please refer to Section 5.5.3 (Predicting cell type using imputed expression of known PBMC marker genes) for details.*

*In brief, we found consistent results between MAST and Wilcoxon. We have also added the following sentence to the manuscript:*

*“The above analyses were performed with MAST, but we found consistent results using the Wilcoxon test (Additional file 1: Figure S5D,E).”*

*Regarding the comment about failures, we have now added a new subsection in Methods to clarify what we mean by “failures”:*

*“4.6.4 Types of failures*

*In the differential expression analysis, there are three types of failures.*

*(1)	“ImputationFail”: It means no imputation results were returned after running the imputation methods for 72 hours. Without imputed values, one cannot proceed with the differential expression analysis. This type of failure is due to failure of the imputation methods, so we assign a zero score to the method, and the score will be counted towards the method’s overall performance in the evaluation and affect the ranking of the imputation methods.*

*(2)	“DifferentialFail”: Here the imputation methods can impute the gene expression, but the methods for detecting differentially expressed genes failed to run. In this scenario, imputation methods themselves did not fail, but one does not have enough information to compare their imputation performance since differential expression analysis cannot be run. Therefore, this type of failure will not be counted in evaluation, and the test results provided by the other test method will be counted only (in our analysis, Wilcoxon rank-sum test can always provide test results).*

*(3) False positives and false negatives: Here both the imputation methods and the differential expression analysis methods (e.g. MAST) can be run without encountering technical issues. However, one may incorrectly identify differentially expressed genes from the imputed data. This further includes two scenarios.*

*(a)	False positives: a non-differential gene is incorrectly reported as differential.*

*(b)	False negatives: a differential gene is incorrectly reported as non-differential.*

*Null differential analysis (Figure 3E-H) specifically evaluates false positives by asking each method to detect differential genes in null analyses where no differential genes are expected. The comparisons to ranking differentially expressed genes (Figure 3A-D) , on the other hand, evaluates each method’s ability to put truly differential genes to top rank which depends on both false positives and false negatives. For a given rank (say top N genes), a better method should have fewer false positives among the top N reported genes and fewer false negatives in the remaining genes. This type of failure will be considered in the evaluation of imputation methods’ overall performance.”*

*We count “ImputationFail” (Type (1)) as the failure of an imputation method. Therefore, when ImputationFail occurs, we assign a zero score to the method, and the score will be counted towards the method’s overall performance in the evaluation and affect the ranking of the imputation methods. “DifferentialFail” (Type (2)), on the other hand, is due to the failure of differential expression methods rather than imputation methods. Therefore, when DifferentialFail occurs, we do not assign a score to the imputation method as it cannot be evaluated, and this failure will not be considered in the evaluation of the imputation methods. For example, for SAUCIE and DeepImpute imputed data, Wilcoxon rank-sum tests can identify differential genes but MAST cannot (please refer to Figure S2). Thus “DifferentialFailure” occurred in the MAST analysis, but not in the Wilcoxon test. As a result, SAUCIE and DeepImpute were not compared in the MAST differential analysis (only the Wilcoxon results of them were counted into the scores in this differential analysis (ranking DEGs), but they were compared in the Wilcoxon differential analysis. This is also the reason why we applied two commonly used differential expression analysis methods in the evaluation. For this reason, the “DifferentialFailure” in Figure S2B for SAUCIE and DeepImpute are not considered as failures of imputation methods and are not be counted in evaluation, because from Figure S2C we can see Wilcoxon test can successfully provide test results.*

\## 2.3 Impact of scRNA-seq imputation on unsupervised clustering

1. Again, it is unclear why the authors used both k-means and Louvain clustering. Figures 4C and F suggests that these two methods give consistent results. How are the two methods different? Do they provide orthogonal metrics for evaluation that provide different information?

*We have now added the following text to the first paragraph in Section 2.3 to explain why we used both k-means and Louvain clustering:*

*“Similar to our motivation for using two DE methods (MAST and Wilcoxon), we considered two unsupervised clustering methods in this section in order to assess whether the performance difference among the imputation methods was due to the choice of the clustering method or if the relative performance of imputation methods was consistent regardless of the choice of clustering method.”*

*k-means is a commonly used centroid-based clustering method, and Louvain is a state-of-the-art graph-based clustering method. Running k-means clustering is fast, and running Louvain clustering is much slower. Louvain clustering was performed by first applying PCA using highly variable genes (HVGs), and then building a shared k-nearest-neighbors (kNN) graph based on the Euclidean distances of the top 10 PCs. An edge was drawn between all pairs of cells sharing at least one neighbor, weighted by the characteristics of the shared nearest neighbors. Clusters were identified by a multi-level modularity optimization algorithm for finding community structure. Louvain clustering is the state-of-the-art in scRNA-seq data analysis, we believe many researchers would be interested to see the evaluation with it, meanwhile since k-means is also efficient and a popular clustering method, we also show the evaluation with it.*

2. The authors use two metrics to assess clustering performance. However, to create an overall score, these four metrics are averaged, which does not seem appropriate given the high correlation between Hacc, Hpur, and ARI (e.g. the metrics are not independent and rather seem redundant). MedianSii also shows opposing trends to the other 3 metrics according to Figure 4.

*We agree with the reviewer on the high correlation of Hacc, Hpur, and ARI and investigated this by calculating the Spearman correlation between all four metrics:*

*To account for this correlation, we have now re-ranked the methods in the clustering section in a new way in which the scores of Hacc, Hour, and ARI are averaged first and then their average is averaged with MedianSil. The Figure 4, Figure 6 and Supplementary Figure S6, S7, and S9 are updated accordingly to match the updated ranking. We found that the overall ranking of methods did not change much.*

*We have also added the following sentence in the Methods (Section 4.11):*

*“For clustering, since H_acc, H_pur and ARI are highly correlated (Figure 4A,D), we first computed the average of H_acc, H_pur and ARI, and then averaged it with medianSil and used the final average as the evaluation score. This is to avoid the three highly correlated metrics (H_acc, H_pur and ARI) dominating the evaluation.”*

3.	The figures refers to entropy of cluster accuracy as Hacc and entropy of cluster purity as Hpur while the main text refers to entropy of cluster accuracy as ECA and entropy of cluster purity as ECP. Please be consistent in your use of acronyms.

*Thank you, we have now modified all ECA with Hacc (entropy of cluster accuracy) and all ECP with Hpur (entropy of cluster purity) to make the main text and figures consistent.*

4.	Based on these performance metrics, MAGIC has consistently the highest overall score. However, in Figure 4E, the UMAP plot from MAGIC shows a large number of tiny spurious clusters and odd 'snakes' that seem to visually suggest that MAGIC is actually performing poorly. In particular, a biologist looking at that MAGIC UMAP plot might be inclined to incorrectly interpret each of those tiny clusters as a distinct cell-type. How have the authors evaluated whether their chosen set of statistics are properly capturing the accuracy of the unsupervised clustering? It may be useful to readers for UMAP plots of outputs from the other imputation methods to also be provided in the supplements.

*We have now added the Additional file 1: Figure S8 which contains UMAP plots of the cells colored by the true cell type, the clustering results from k-means, and the clustering results from Louvain clustering. The spurious shapes of UMAP can be seen in quite a few imputation methods in addition to MAGIC (kNN-smoothing, DCA, SAUCIE, SAUCIE_latent, scScope).*

*The problem that the reviewer describes is mainly due to UMAP visualization rather than imputation and clustering. Our clustering is done on principal components (PC) space using top 10 PCs rather on the 2 dimensional UMAP coordinates in this paper for all the imputation methods including MAGIC. Principal component analysis is a linear transformation of the original data itself and is less likely to produce spurious signals by UMAP. Also, when projecting a higher dimensional data (e.g. 10-dimensional PC) to a lower dimensional space (2-dimensional UMAP), information will be lost. Thus, the UMAP visualization does not necessarily reflect clustering accuracy. We have observed this phenomenon in many of our other projects. The clustering based on PCA is able to differentiate major cell types, indicating that the clustering can indeed capture the underlying biological signal quite well, as shown in the following barplot, which shows the proportion of cell types classified in each cluster in Figure 4E (right-hand-side UMAP for MAGIC).*

\## 2.4 Impact of scRNA-seq imputation on inferring pseudotemporal trajectories

1. The authors expand the analysis here to 21 scRNA-seq imputation methods. What are these 3 new scRNA-seq imputation methods now included (they are not explicitly named in the text)? It is also unclear why this choice was made; why were previous analyses not performed with all 21 scRNA-seq imputation methods? Were the 3 new scRNA-seq imputation methods specific for pseudotemporal trajectory inference?

*We thank the reviewer for pointing this out. Previously, we were not clear in what we meant. We clarify it here and in the revised manuscript in Sections 2.3, 2.4 and 4.7.*

*There are a total of 18 imputation methods we evaluated in this benchmark. However, among these 18 methods, three of them (scVI, SAUCIE, scScope) are deep-learning methods that output both*

*(1)	the latent space representation of the cells (only used in Figure 1, 4, 5)*

*(2)	the imputed gene expression profiles of the cells (e.g. used in all the figures).*

*Intuitively, the latent space representation can be viewed as projecting the cells to a low dimensional space. In the low-dimensional space, you only keep cell-to-cell distances (i.e. dissimilarities) and do not keep the expression value of each gene. Therefore, one cannot use the latent space representation to perform gene-level analysis such as comparing how well imputed gene expression values correlate with bulk gene expression profile, or comparing how well one can detect differentially expressed genes. That is why the latent space representations generated by the three deep-learning methods were not used in our Figure 2 (similar to bulk analysis) and Figure 3 (differential expression analysis). However, one can use the latent space representation to study cells’ relationship such as cell clustering and cells’ progressive change along pseudotime. That is why these 3 latent representations are used in the cell clustering and pseudotime analyses in Figures 4-5.*

*Specifically, the combination of the 18 imputation methods plus the 3 latent spaces from the imputation methods (scVI, SAUCIE, scScope) -- for a total of 21 -- are used in Figures 4-5. Therefore, for these three methods, one can either use the latent space representation directly for clustering cells, or use the imputed gene expression profiles to cluster the cells and construct pseudotemporal trajectories. Previously we referred to these as different “methods”, but in fact they are the same method; we are just using a different output from the same method and refer to the different approaches as scVI, scVI_latent, SAUCIE, SAUCIE_latent, scScope, and scScope_latent (for example see Additional file 1: Figure S8).*

*We have modified the text in the manuscript in paragraph 1 in Section 2.3:*

*“However, instead of only considering the imputed gene expression profiles from the 18 imputation methods, we also considered the three latent spaces from scVI, scScope and SAUCIE (for a total of 21 “methods”) for unsupervised clustering (Section 2.3) and inferring pseudotemporal trajectories (Section 2.4).”*

*We have also added added a sentence in Methods Section 4.7 (Evaluation of imputation on unsupervised clustering):*

*“For scVI_latent, scScope_latent, and SAUCIE_latent, we skipped the principal component analysis of the imputed values and replaced the top 10 PCs using the latent space coordinates obtained from scVI, scScope, and SAUCIE.”*

2. In Figure 6, the heatmap includes time, memory, and scalability. It is unclear what scale time and memory are on.

*We have now clarified this by incorporating this information into the legend of Figure 6:*

*“Performance of imputation methods in all evaluation aspects: similarity between imputed single-cell and bulk profiles (denoted as “bulk correlation”), differential expression (“differential”), unsupervised clustering (“clustering”), trajectory inference (“trajectory”), time, memory-usage and scalability. The units of computational time (in minutes), memory usage (in maximum resident set size of all tasks in job (MaxRSS or maximum resident set size of all tasks in a job) in gigabytes (GB), and scalability (w.r.t. the number of observations or cells) were all scaled to be in [0,1] to apply the same color scale. For more details on units of computational time, memory usage and scalability, see Section 4.10 and details in Additional file 1: Figure S11. A higher score represents a better performance. Imputation methods are ranked by averaging the scores in bulk correlation, differential, clustering, and trajectory. No imputation is abbreviated as “no_imp". (A) Performance scores and the ranking of all imputation methods using datasets across UMI-based and Plate-based (Fluidigm) protocols; (B) using UMI-based data only; (C) using Fluidigm data only. (D) Practical guidelines for method users. For users who prefer imputation accuracy (UMI-based and plate-based rows in the table), we listed methods that perform better than no imputation for each analysis task. For each task, methods with better performance are listed on top, and the top five methods are displayed in color. For users who prefer computational efficiency (last row in the table), we first obtained methods that were ranked in top 50% in terms of overall time, memory and scalability performance. Then for each analysis task, we retained methods that outperformed no imputation in terms of accuracy, and we listed these methods in the table in the rank order based on their overall accuracy performance (averaged across UMI- and plate-based platforms) in each analysis.”*

*We also incorporated the units into the text in Section 4.10:*

*“By running the imputation methods on all datasets, we assessed the computational time (in minutes), memory usage (MaxRSS or maximum resident set size of all tasks in a job in gigabytes (GB)) returned from the Slurm command sacct), and scalability with respect to cell number (Additional file 1: Figure S11, Additional file 6: Table S5).”*

\## Conclusion/Overall

1.	The authors conclude that the performance of these imputation methods depends on many factors including the experimental protocol, the sparsity of the data, the number of cells in the dataset, and the magnitude of the effect sizes. But it is not clear how these factors, either individually or in combination, impact each imputation method. For example, if ! am using the Fluidigm system with a few cells but am looking for large magnitude effect sizes, which method should ! use? Or if ! am using the 10X system with millions of cells but high sparsity, which method should ! use? Or is no imputation the safest choice?

*Thank you very much for your comments. We agree that it is necessary to make it more clear how different factors could affect the selection of an imputation method. To address this, we now provide a flow chart in the new Figure 6D in the main manuscript.*

2.	How do the authors' findings compare to Tian et al (mentioned in the paper) and other benchmarking analyses such as Andrews and Hemberg (2019) or Zhang and Zhang (2018)? !f there are inconsistencies between these different benchmarking papers, the authors need to discuss why these inconsistencies arise and why their benchmarking provides a more valid performance assessment for these imputation methods.

If they are highly consistent, the authors need to justify why their benchmarking represents a significant advance over these previously published studies.

*We have now added Additional file 2: Table S1 to summarize how our results compare to the Tian et al. (2019), Zhang and Zhang (2018), and Andrews and Hemberg (2019) benchmarking analyses. We provide a figure below of the CSV file provided in the supplement (Additional file 2: Table S1).*

*To briefly summarize, our benchmark study is: (1) the one that include the most comprehensive set of imputation methods in which many new high-profile methods are included, for example scVI, SAVER-X; (2) the only one that systematically evaluates scRNA-seq imputation using cell lines and tissue data across both droplet-based and plate-based protocols in both similarity to bulk profiles and downstream analysis; and (3) the only one that provides a clear score for readers to navigate the imputation methods so as to choose the one that is most suitable for their data and their analysis.*

*We have now added this sentence to the Introduction section:*

*“Compared to existing benchmark evaluations of imputation methods, our benchmark is more comprehensive in terms of (i) the number of imputation methods considered (18 imputation methods compared to 3-8 in previous benchmarks [13, 14, 18]), (ii) the types of tissues considered (both cell lines and tissues), (iii) the number of downstream analyses considered (four applications compared to just one, for example, differential expression [14]), and (iv) different types of experimental protocols considered (both droplet- and plate-based protocols as opposed to just plate-based [13]), all of which is described in Additional file 2: Table S1.”*

*Finally, we have added a table summarizing these results in Additional file 2: Table S1 (shown below here for review).*

3. While this topic remains of outstanding interest to a broad audience of biologists, the way the paper is currently written would be difficult for such a broad audience of biologists to understand. Many of the analyses would greatly benefit from further clarification as to the motivation for the analysis, why certain choices were made in terms of metrics/statistics, and a more in-depth interpretation of the findings.

*Thank you for the helpful comments. We hope that with the revisions enclosed and described above have clarified many of the confusions you have raised.*

Reviewer #2 :

Accept with minor revisions (no re-review needed IMHO) because the field needs to see this paper -- a null simulation shows off how many methods are really just geared to make garbage data "look compelling". I was a little disappointed that the authors did not touch on RNA velocity, since both trajectory and transcriptome inference at present largely focus on the summed abundance of spliced and unspliced RNA -- this is particularly dumb since the correlation of a given cell at a given time point with its neighbors will be dominated by the spliced abundance, whilst the unspliced abundance (and more importantly, the ratio of the two) is almost certainly a better indicator of the cell's fate. However, integrating data from spliced and unspliced separately decouples this relationship, and as far as I know, nobody has really tried to separate the dual projections and reconcile them. So the idea of swapping out correlation-based trajectories for velocity-based trajectories ends up driving full speed into the brick wall of an open research problem.

As a bonus, without either spike-in cells (per the recent paper from Christoph Bock and colleagues) or spike-in RNA molecules representing both spliced and unspliced transcripts (per the recent SmartSeq3 paper), I'm not sure any of these things can be considered quantitative. (Trajectories in general can be thought of as a depiction of where the cells were perhaps 6 hours ago, which is a bit perverse considering how fast things like Wnt/Hedgehog planar cueing, JAK-STAT signaling, steroid receptors, or metabolic rewiring happen!)

*We thank the reviewer for the positive and helpful comments. While RNA velocity is an exciting application, we consider this outside the scope of the manuscript. However we have now incorporated the following in the Discussion and Conclusions section:*

*“An open problem not investigated in our current study is the impact of imputation methods on the RNA velocity analysis [57, 58, 59]. Since RNA velocity is estimated by analyzing unspliced and spliced mRNA, it takes into account both spliced and unspliced counts. Existing imputation methods deal with the drop-out events by imputing the gene expression values rather than the original reads, and the gene expression is usually quantified on exons only which may not distinguish contributions from the spliced versus unspliced transcripts. Therefore, whether existing imputation methods can also be applied to velocity analysis and to separately impute spliced and unspliced transcripts (including introns) remains an open problem that requires extensive future investigation which is beyond the scope of the present study. In addition to the velocity analysis, evaluating how imputation methods may impact other emerging analyses such as spatial transcriptomics [60, 61, 62, 63, 64] also warrants future investigation.*

But at the end of the day, this paper shows that most imputation methods are useless for deep inference *even when they successfully complete matrices equivalent to bulk* and neither improve lineage inference nor clustering, perhaps the two most common tasks in single-cell RNA sequencing analyses. I suspect the reason is very deep-seated and requires more thought than many groups seem willing to give it; happily, neither Dr. Hicks nor Dr. Ji's lab has ever been shy about tackling brutally difficult, unpopular problems (cf. BIRD).

*We really appreciate your very positive comments on our work. Our study indicates that the development of scRNA-seq imputation methods is still far from mature, particularly in analyses that require the information of cell topology, such as clustering, trajectory and network analysis. We agree with you that the reason could be very deep-seated and better imputation methods are anticipated.*

I think this paper is of great interest to all readers who care about science instead of science fiction; both Ben Johnson and Pamela Himadewi in my lab agree.

*We again would like to express that we greatly appreciate your very positive comments.*

**Second round of review**

**Reviewer 1**

The authors have done a thorough job addressing my concerns. One additional point of concern is that the benchmarks performed relied primarily on data from stable cell lines and from healthy patients. A brief discussion on how these results may or may not translate to applications in a diseased setting such as cancer would be greatly appreciated.

**Authors’ Point-by-point response to reviewers**

Reviewer #1: The authors have done a thorough job addressing my concerns. One additional point of concern is that the benchmarks performed relied primarily on data from stable cell lines and from healthy patients. A brief discussion on how these results may or may not translate to applications in a diseased setting such as cancer would be greatly appreciated.

*We have added another few sentences to our discussion (see comments from Reviewer #1)*

*“Another limitation is that there are no disease tissues included in this study. In the future, it is worthwhile to continue investigating how conclusions presented in this study may translate to applications in a diseased setting such as cancer tissues. One challenge is that the expression of some genes in diseased cells might be abnormal [64, 65, 66, 67, 68], which could lead to the false identification of similar cells and could affect the imputation performance.”*
